# Flavonoids in cancer pharmacology: molecular mechanisms, tumor microenvironment regulation, and drug resistance

**DOI:** 10.3389/fphar.2026.1969337

**Published:** 2026-10-12

**Authors:** Zhengyuan Yu, Zhanyi Zhou, Ni Yin, Hongcheng Ge

**Affiliations:** 1 Department of Medical Oncology, The First Affiliated Hospital of Soochow University, Suzhou, China; 2 The First School of Clinical Medicine, Zhejiang Chinese Medical University, Hangzhou, China; 3 The First Affiliated Hospital of Zhejiang Chinese Medical University (Zhejiang Provincial Hospital of Traditional Chinese Medicine), Hangzhou, China

**Keywords:** drug resistance, epithelial–mesenchymal transition, flavonoids, metabolic reprogramming, regulated cell death, tumor microenvironment

## Abstract

Flavonoids are structurally diverse polyphenolic compounds that influence multiple processes involved in cancer development and treatment response. This review summarizes the reported effects of structurally defined flavonoids on tumor cell proliferation, regulated cell death, oncogenic and epigenetic signaling, metabolic adaptation, the tumor microenvironment, angiogenesis, and therapy resistance. PubMed, Web of Science, and Scopus were searched for literature published primarily from 2016 to July 2026 using terms related to flavonoids, representative flavonoid compounds, cancer-associated mechanisms, drug resistance, combination therapy, drug delivery, pharmacokinetics, bioavailability, and safety. Earlier landmark and mechanistic studies were included when necessary to provide historical context or support established pathways. Experimental evidence indicates that flavonoids can modulate cell cycle progression, apoptosis and other regulated cell death pathways, inflammatory and immune signaling, tumor metabolism, drug efflux transporters, epithelial to mesenchymal transition, and epigenetic programs associated with resistance. Their effects are nevertheless dependent on compound structure, dose, tumor model, cellular redox state, and treatment context. Combination and nanodelivery strategies may improve experimental activity or exposure, but their interpretation requires consideration of quantitative drug interactions, active metabolites, pharmacokinetic changes, and safety. Most available evidence remains preclinical, and clinical translation is constrained by limited bioavailability, extensive metabolism, heterogeneous tumor responses, and possible interactions with anticancer drugs. Further development should therefore emphasize chemically defined compounds, clinically achievable exposure, functional validation of proposed mechanisms, standardized formulations, and integrated pharmacokinetic and safety assessment.

## 1 Introduction

Cancer is characterized by uncontrolled cell growth, dysregulated signaling, and multiple forms of adaptive escape. Its development is driven by genetic mutations, epigenetic alterations, metabolic reprogramming, and persistent remodeling of the tumor microenvironment (Hanahan, 2022; Kaleem et al., 2025). Targeted therapy and immunotherapy have improved outcomes in selected cancers. However, durable responses remain difficult to achieve because of tumor heterogeneity, compensatory survival signaling, immune evasion, and therapy resistance (Li et al., 2025). These limitations support the continued search for pharmacological agents that regulate multiple cancer-associated pathways rather than a single molecular target.

Natural products remain an important source of pharmacologically active small molecules because they often act on multiple targets rather than a single pathway (Efferth et al., 2020; Liu et al., 2020). In cancer research, alkaloids, terpenoids, flavonoids, quinones, and steroidal compounds are structurally diverse and biologically active (Simoben et al., 2015; Nwodo et al., 2016). Many of these compounds affect tumor cell proliferation, survival, apoptosis, and therapy response through regulation of cancer-related signaling pathways (Efferth et al., 2020; Nwodo et al., 2016; Simoben et al., 2015). Their multi-pathway activity may help address limitations of highly selective single-target therapies (Renault-Mahieux et al., 2022).

Flavonoids are polyphenolic secondary metabolites widely found in fruits, vegetables, tea, and medicinal plants (Kopustinskiene et al., 2020; Wen et al., 2020). Most flavonoids share a benzo-γ-pyrone backbone with two aromatic rings and one heterocyclic pyran ring, whereas variations in hydroxylation, glycosylation, methylation, and ring substitution contribute to their diverse biological activities (Havsteen, 2002; Kumar and Pandey, 2013; Kuljarusnont et al., 2024). In cancer models, flavonoids can inhibit proliferation, trigger regulated cell death, interfere with oncogenic signaling, alter metabolic adaptation, reshape the tumor microenvironment, and modulate therapy resistance (Pang et al., 2021; Zhou Z. G. et al., 2022; Xu et al., 2024). Recent epidemiological evidence also links higher flavonoid intake with lower all-cause mortality in cancer patients, although these observations do not establish therapeutic efficacy (Jia et al., 2025).

Here, pharmacological regulation at the network level refers to the ability of a defined compound to perturb multiple connected targets or pathways that influence tumor cell, stromal, or immune phenotypes. This concept does not imply direct binding to every affected molecular node. The present review focuses primarily on structurally defined flavonoids, while chemically characterized fractions enriched in flavonoids are included only as supportive context. We summarize their reported effects on cancer-associated signaling, regulated cell death, metabolic adaptation, the tumor microenvironment, and therapy resistance, together with emerging combination and delivery strategies and the principal barriers to clinical translation (Figure 1; Table 1).

**FIGURE 1:**
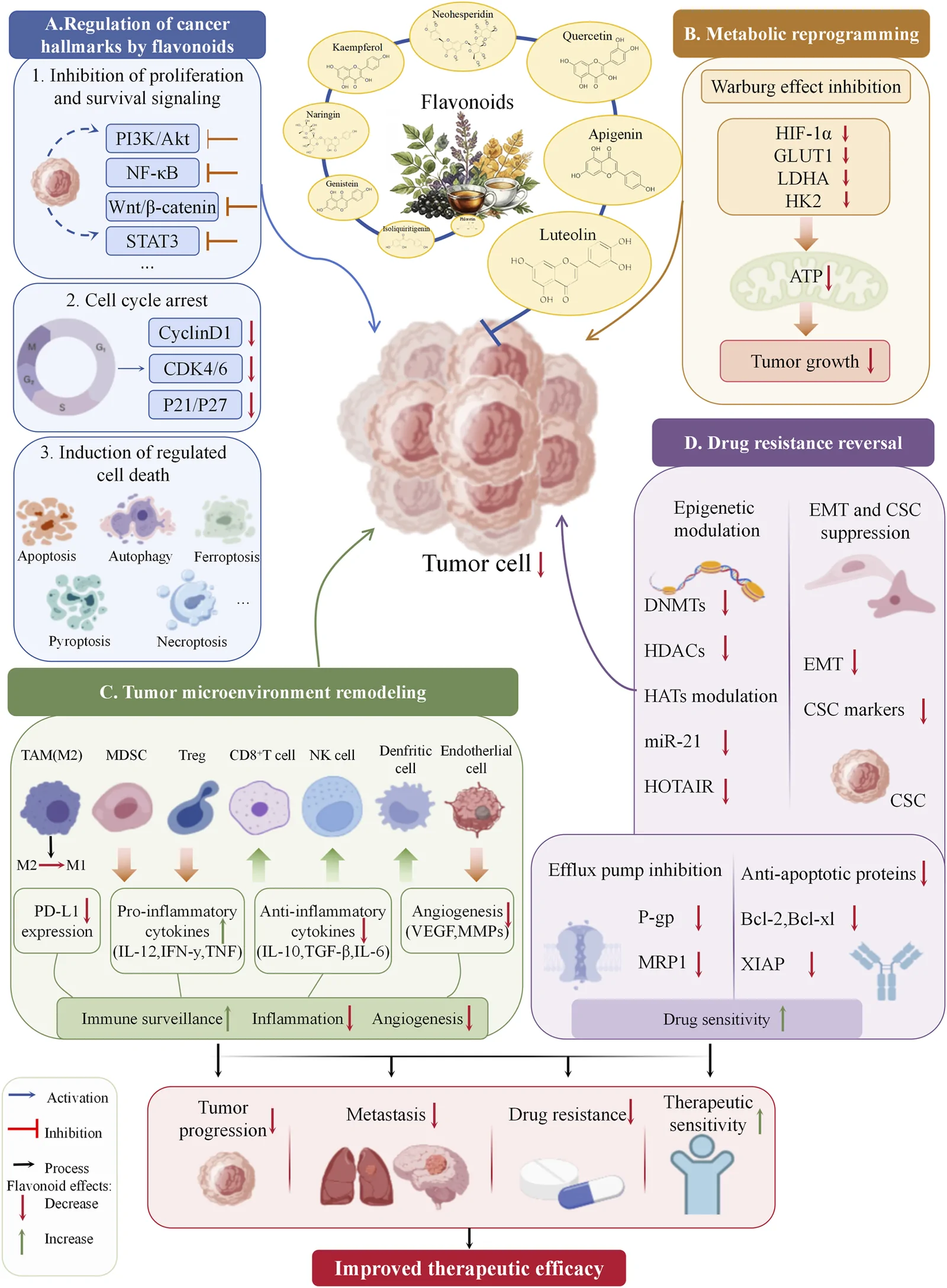
Multi-target pharmacological mechanisms of flavonoids in cancer. **(A)** Regulation of cancer hallmarks in tumor cells, including inhibition of proliferation, induction of cell-cycle arrest, and regulated cell death. **(B)** Metabolic reprogramming. **(C)** Tumor microenvironment remodeling. **(D)** Drug resistance reversal. Created with BioGDP.com (Jiang et al., 2025).

**TABLE 1:** Mechanisms of flavonoids in cancer therapy.

Flavonoid	Cancer type	Concise mechanism	Evidence level	References
Apigenin	Breast cancer	Inhibits PI3K/Akt–FOXO3a and induces p21/p27-mediated cell cycle arrest	*In vitro*	Lin et al. (2015)
	Colorectal cancer	Suppresses Wnt/β-catenin signaling and inhibits proliferation and invasion	*In vitro*	Xu et al. (2016)
	Prostate and breast cancer	Blocks IKKα/NF-κB signaling and inhibits HDAC-mediated tumor growth	*In vitro* + *in vivo*	Shukla et al. (2015), Pandey et al. (2012), Tseng et al. (2017)
	Pancreatic and renal cancer models	Activates p53/p21-associated signaling and induces cell cycle arrest and apoptosis	*In vitro*	King et al. (2012), Bao et al. (2022)
	Ovarian cancer/NSCLC	Downregulates HIF-1α/VEGF and suppresses angiogenesis	*In vitro* + angiogenesis assay	Fang et al. (2007), Fu et al. (2022)
	Melanoma	Reduces STAT1 phosphorylation and IFN-γ-induced PD-L1 expression combined with curcumin	*In vitro* + *in vivo*	Xu et al. (2018)
	Multiple p53-competent cancer cell models	Enhances cisplatin-induced DNA damage and apoptosis through MAPK-dependent p53 phosphorylation and accumulation	*In vitro*; combination evidence	Liu et al. (2017)
	Cisplatin-induced renal injury model	Attenuates renal oxidative stress, inflammation, and functional injury in non-tumor-bearing mice	*In vivo* toxicity model; not an antitumor-efficacy study	Hassan et al. (2017)
Luteolin	Breast cancer	Inhibits PI3K/Akt signaling and promotes apoptosis	*In vitro*	Lin et al. (2015)
	Pancreatic cancer	Suppresses NF-κB/STAT3 signaling and enhances gemcitabine efficacy	*In vitro* + *in vivo*	Johnson et al. (2015)
	Head and neck squamous cell carcinoma	Inhibits p300 lysine acetyltransferase activity and suppresses tumor growth	*In vitro* + *in vivo*	Selvi et al. (2015)
	Lung cancer	Reduces CCL2, EMT, and TAM-driven angiogenic signaling	*In vitro*/co-culture	Choi et al. (2016), Fang et al. (2018), Ruan et al. (2012)
	Clear cell renal cell carcinoma	Increases the labile iron pool through excessive HO-1 activity and promotes glutathione depletion and lipid peroxidation; ferroptotic death is partially rescued by deferiprone or ferrostatin-1	*In vitro* + *in vivo*; pharmacological rescue	Han et al. (2022)
	Prostate cancer	Promotes TFEB-dependent ferritinophagy and iron-dependent lipid peroxidation, supported by TFEB knockdown and pharmacological rescue	*In vitro* + *in vivo*; genetic/pharmacological validation	Fu et al. (2024)
Quercetin	Hepatocellular carcinoma	Regulates ROS, p53/HO-1, Cyclin A, and CHK1 to inhibit proliferation	*In vitro*	Jeon et al. (2019)
	Lymphoma	Inhibits PI3K/Akt/mTOR and STAT3, inducing apoptosis and autophagy	*In vitro*	Granato et al. (2017)
	Breast/prostate cancer	Suppresses glycolysis, stemness, and migration-related signaling	*In vitro*	Erdogan et al. (2018), Jia et al. (2018)
	Breast cancer	Promotes TFEB nuclear translocation and lysosomal ferritin degradation, leading to iron release and lipid peroxidation; TFEB silencing or chloroquine attenuates the response	*In vitro*; genetic/pharmacological validation	An and Hu (2022)
	Doxorubicin-resistant breast cancer	Co-encapsulation with doxorubicin in biotin-modified PEG–PCL nanoparticles enhances intracellular drug retention and suppresses P-gp and PI3K/Akt signaling	*In vitro* + *in vivo*; co-delivery model	Lv et al. (2016)
	Cervical cancer	Inhibits DNMT activity and reduces aberrant promoter methylation, contributing to reactivation of tumor-suppressor genes	*In vitro*	Kedhari Sundaram et al. (2019)
	Prostate cancer/osteosarcoma	Modulates oncogenic miRNAs and enhances chemotherapy sensitivity	*In vitro*	Yang et al. (2015), Zhang X. et al. (2015)
	5-FU-resistant colorectal cancer	Enhances the response to 5-FU by suppressing treatment-induced thymidylate synthase expression and promoting p53-associated mitochondrial dysfunction	*In vitro*; resistant-cell model	Yang C. et al. (2022)
Isorhamnetin	Pancreatic cancer-associated fibroblasts	Activates p53–p21 and induces G2/M arrest	*In vitro*	Ganbold et al. (2025)
Naringenin	Colorectal cancer	Activates p38-dependent Cyclin D1 degradation and induces G1 arrest	*In vitro*	Song et al. (2015)
		Naringenin-loaded nanostructured lipid carriers enhance oxaliplatin-associated apoptosis and cell cycle inhibition	*In vitro*; nanodelivery combination model	Raeisi et al. (2019)
	Breast cancer pulmonary metastasis model	Reduces TGF-β1 secretion and suppresses Treg/MDSC-mediated immune suppression	*In vitro* + *in vivo*	Zhang et al. (2016)
	Hepatocellular carcinoma	Enhances sensitivity to ferroptosis inducers by activating AMPK–PGC-1α signaling and suppressing aerobic glycolysis	*In vitro*; quantitative combination and pathway-inhibition evidence	Li et al. (2024)
Naringin	Triple-negative breast cancer	Inhibits β-catenin signaling and promotes G1 arrest and apoptosis	*In vitro*	Li et al. (2013)
	Colon cancer	Suppresses HIF1A/ENO2 signaling and inhibits aerobic glycolysis	*In vitro*	Pan et al. (2023)
	Melanoma	Inhibits c-Src/HIF-1α signaling and metabolic adaptation	*In vitro*	Guo et al. (2016)
Neohesperidin	Osteosarcoma	Activates ROS/JNK signaling and induces G2/M arrest, apoptosis, and autophagy	*In vitro*	Wang S. et al. (2021)
Fisetin	Non-small cell lung cancer	Blocks EMT and reduces cancer stem cell-like traits	*In vitro*	Tabasum and Singh (2019)
	Prostate cancer	Inhibits mTOR signaling and induces autophagy-related cell death	*In vitro*	Suh et al. (2010)
	Melanoma/skin cancer	Induces ER stress-associated apoptosis and suppresses melanoma growth through direct inhibition of mTOR and p70S6K	*In vitro* + 3D melanoma model + xenograft	Syed et al. (2014a), Syed et al. (2014b), Kubina et al. (2022)
Phloretin	Hepatocellular carcinoma	Activates SHP-1 and inhibits STAT3/AKT/mTOR/ERK signaling	*In vitro* + *in vivo*	Saraswati et al. (2019)
	Sorafenib-resistant hepatocellular carcinoma	Enhances the antitumor activity of sorafenib by activating SHP-1 and suppressing STAT3-associated survival signaling	*In vitro* + *in vivo*; resistant-cell combination model	Saraswati et al. (2019)
Isoliquiritigenin	Melanoma	Inhibits STAT3 phosphorylation and downregulates PD-L1	*In vitro* + *in vivo*	Zeng et al. (2025)
	Hepatocellular carcinoma	Suppresses NF-κB survival signaling, reduces Bcl-xL and c-IAP1/2 expression, and induces caspase-dependent apoptosis	*In vitro*	Hsu et al. (2005)
	Melanoma	Inhibits STAT3 phosphorylation and PD-L1 expression and attenuates tumor progression in melanoma models	*In vitro* + *in vivo*	Zeng et al. (2025)
	Bladder cancer	Disrupts mitochondrial membrane potential and activates Bax/Bim–Apaf-1–caspase-9/caspase-3 signaling, resulting in mitochondrial apoptosis	*In vitro*	Si et al. (2017)
	Colitis-associated cancer	Blocks M2 macrophage polarization through PGE2/PPAR-δ and IL-6/STAT3	*In vivo* + mechanistic validation	Zhao et al. (2014)
Total flavonoid aglycones from Scutellaria baicalensis	Non-small cell lung cancer	Induces S-phase arrest and suppresses xenograft tumor growth	*In vitro* + *in vivo*; flavonoid-enriched fraction	Wang et al. (2016)
Genistein	Colon cancer	Suppresses Wnt/β-catenin activation and colorectal preneoplasia	*In vivo*	Zhang et al. (2013)
	Renal cancer	Downregulates miR-1260b and restores Wnt antagonist expression	*In vitro*	Hirata et al. (2013)
	Prostate/breast cancer	Modulates DNA methylation and chromatin-associated regulation of BRCA1/2, ERβ, p21, and p16	*In vitro*/epigenetic evidence	Bosviel et al. (2012), Majid et al. (2008), Mahmoud et al. (2015)
	Prostate cancer	Upregulates miR-34a and suppresses oncogenic HOTAIR, contributing to inhibition of tumor cell growth	*In vitro*; xenograft validation of HOTAIR function	Chiyomaru et al. (2013)
Kaempferol	Hepatocellular carcinoma	Inhibits p44/42 MAPK-dependent nuclear accumulation and transcriptional activity of HIF-1α under hypoxic conditions, thereby reducing hepatoma-cell viability	*In vitro*	Mylonis et al. (2010)
	Pancreatic cancer	Inhibits EGFR-related signaling and suppresses tumor growth	*In vitro*	Lee and Kim (2016)
	Colorectal cancer	Synergizes with 5-FU through PI3K/Akt regulation	*In vitro*	Li Q. et al. (2019)
	Endothelial cells	Inhibits VEGFR2/PI3K/Akt signaling and angiogenesis	*In vitro* angiogenesis model	Chin et al. (2018)
Puerarin	Colon cancer	Inhibits PI3K/Akt signaling and suppresses EMT, migration, and invasion	*In vitro*	Chen et al. (2025)
Anthocyanins	Colorectal cancer	Upregulate KLF4 and inhibit ERK/p38 signaling	*In vitro* + *in vivo*	Chang and Xiao (2025)
	Glioma	Suppress TGF-β/Smad signaling and reverse EMT	*In vitro*	Ouanouki et al. (2017)
Baicalein	Breast cancer	Inhibits PI3K/Akt signaling and induces apoptosis and autophagy	*In vitro* + *in vivo*	Yan et al. (2018)
	Lung cancer	Activates AMPK/ULK1 and Drp1-mediated mitochondrial death pathways	*In vitro*	Deng et al. (2020), Aryal et al. (2014)
	Gastric cancer	Suppresses PI3K/Akt/HIF-1α glycolytic signaling and reverses 5-FU resistance	*In vitro*	Chen et al. (2015)
	Broad tumor models	Regulates redox-related death pathways, including autophagy and ferroptosis-related responses	*In vitro*	Vetrivel et al. (2019), Deng et al. (2020), Aryal et al. (2014)
	Hepatocellular carcinoma	Suppresses STAT3 activity and PD-L1 expression	*In vitro*	Ke et al. (2019)
	Tumor-associated macrophages	Targets PI3Kγ/NF-κB to promote M1 polarization and inhibit M2 polarization	*In vitro*/co-culture	He et al. (2021)
	Paclitaxel-resistant breast cancer	Co-delivery with paclitaxel in nanoemulsions enhances antitumor activity by increasing oxidative stress and reducing P-gp-associated drug efflux	*In vitro* + *in vivo*; co-delivery model	Meng et al. (2016)
Wogonin	Multiple myeloma	Promotes HIF-1α degradation and suppresses VEGF/PDGF/bFGF secretion	*In vitro* + *in vivo*	Fu et al. (2016)
	Tumor immune microenvironment	Regulates TGF-β1/Smad signaling and Treg differentiation	*In vitro*/co-culture	Dandawate et al. (2012)
Scutellarin	Colon cancer model	Blocks TNF–TNFR2 signaling and increases CD8^+^ T-cell infiltration	*In vitro* + *in vivo*	Chen et al. (2022)
Hesperidin	Breast cancer	Inhibits AKT/NF-κB signaling and downregulates PD-L1	*In vitro*	Kongtawelert et al. (2020)
Icaritin	Solid tumor models	Targets IKK-α/NF-κB signaling and reduces PD-L1 expression	*In vitro* + *in vivo*	Mo et al. (2021)
EGCG	Gastric cancer	Regulates the LINC00511/miR-29b/KDM2A axis and suppresses progression	*In vitro*	Zhao et al. (2020)
	Breast/esophageal/lung cancer	Inhibits DNMT1 and reactivates tumor suppressor genes	*In vitro*	Sheng et al. (2019), Meng et al. (2017), Gao et al. (2009)
	Tamoxifen-resistant breast cancer	Suppresses Nrf2-associated antioxidant signaling and partially restores tamoxifen responsiveness	*In vitro*; resistant-cell model	Esmaeili (2016)
Myricetin	Hepatocellular carcinoma	Induces autophagy and cell cycle arrest by suppressing MARCH1 and modulating STAT3 and p38 MAPK signaling	*In vitro* + *in vivo*	Yang et al. (2021)
	Hepatocellular carcinoma, including cisplatin-response models	Activates LATS1/2-dependent phosphorylation and degradation of YAP, thereby inhibiting proliferation and enhancing the response to cisplatin	*In vitro* + xenograft	Li M. et al. (2019)
Silibinin	Breast cancer	Induces BNIP3-associated autophagy through ROS-dependent mitochondrial dysfunction, loss of mitochondrial membrane potential, and ATP depletion	*In vitro*	Jiang et al. (2015)
	Prostate cancer	Reduces the expression of PRC2 components EZH2, SUZ12, and EED and decreases HDAC1/2 expression, while producing a context-dependent increase in global H3K27me3	*In vitro*	Anestopoulos et al. (2016)
	EGFR-mutant or ALK-rearranged non-small cell lung cancer	Enhances responses to selected EGFR or ALK tyrosine kinase inhibitors by suppressing EGFR-family signaling, persistent STAT3 activation, and EMT-associated TGF-β/SMAD signaling	*In vitro* + xenograft/preclinical evidence	Rho et al. (2010), Cuyàs et al. (2016), Verdura et al. (2022)
3,6-Dihydroxyflavone	Breast cancer	Suppresses TGF-β-induced EMT, possibly through Notch-related signaling	*In vitro*	Chen et al. (2016)
Quercetin, apigenin, luteolin, kaempferol, baicalein, and related flavonoids	Multidrug-resistant cancer models	Modulate P-gp-mediated transport through substrate-pocket interaction, altered ATPase activity, or reduced transporter expression; the strength of evidence varies among compounds	Biochemical, transport-assay, and preclinical evidence	Conseil et al. (1998), Bai et al. (2019), Fang et al. (2021), Ye et al. (2019)

### 1.1 Literature search and evidence synthesis

A structured narrative literature search was conducted in PubMed, Web of Science, and Scopus. The review focused primarily on studies published from January 2016 to July 2026, and the literature search was last updated in July 2026. Earlier landmark studies were also considered when needed to describe established pathways, foundational pharmacological concepts, or the historical development of the field. Search terms included flavonoid class names and the names of representative compounds discussed in the manuscript, such as apigenin, luteolin, quercetin, kaempferol, naringenin, baicalein, genistein, fisetin, and related derivatives. These terms were combined with cancer- or tumor-related concepts covering proliferation and cell-cycle regulation, regulated cell death, oncogenic signaling, epigenetics and non-coding RNA, metabolic reprogramming, the tumor microenvironment, immune regulation, angiogenesis, drug resistance, combination therapy, drug delivery, pharmacokinetics, bioavailability, and safety. Reference lists of relevant studies and reviews were also examined to identify additional publications.

Studies were selected when they examined structurally defined flavonoids or sufficiently characterized flavonoid derivatives and reported findings relevant to cancer biology, anticancer activity, treatment response, molecular mechanisms, pharmacokinetics, drug delivery, or safety. The review considered biochemical and target-based investigations, conventional and advanced *in vitro* models, animal studies, resistant-tumor models, combination-treatment studies, pharmacokinetic studies, and available human or epidemiological evidence. Relevant reviews were used to provide background and identify additional primary studies. Chemically undefined extracts, duplicate reports, studies without cancer-relevant outcomes, and conclusions based only on molecular docking or isolated marker changes were not treated as primary mechanistic evidence. Studies of chemically characterized flavonoid-enriched fractions were retained only as supportive evidence.

Because the included literature differed substantially in flavonoid structure, cancer type, experimental model, dose, formulation, exposure duration, and measured outcome, the evidence was synthesized qualitatively rather than by meta-analysis. The principal studies were organized by flavonoid, cancer type, concise mechanism, and level of experimental support, as summarized in Table 1. Evidence levels included biochemical or target-based findings, conventional *in vitro* studies, advanced *in vitro* or coculture models, animal studies, combined *in vitro* and *in vivo* evidence, resistant-cell or combination-treatment models, and human evidence where available. Greater interpretive weight was given to studies that combined multiple experimental systems or used direct target assays, pathway perturbation, rescue experiments, pharmacological inhibition, genetic manipulation, quantitative combination analysis, or pharmacokinetic measurements. This framework was used to distinguish preliminary marker-based observations from findings supported by functional or multi-model validation.

## 2 Cancer hallmarks and the regulatory effects of flavonoids

### 2.1 Abnormal proliferation and cell-cycle regulation

Enhanced proliferative capacity is a defining feature of cancer and reflects disruption of the cell cycle regulatory network (Hanahan, 2022). In normal cells, cyclins, cyclin-dependent kinases (CDKs), and CDK inhibitors (CKIs) coordinate orderly progression through the G1, S, G2, and M phases (Wang, 2021). In tumor cells, this balance is often lost. Cyclin D1, Cyclin E, and CDK4/6 are frequently activated, whereas inhibitory regulators such as p21 and p27 are reduced or functionally impaired (Sanchez-Vega et al., 2018). This imbalance allows tumor cells to evade growth control and maintain continuous proliferation (Sanchez-Vega et al., 2018).

Accumulating evidence indicates that flavonoids interfere with cell cycle control and suppress tumor growth (Zhou Z. G. et al., 2022). In breast cancer cells, flavone, apigenin, and luteolin reduce cell viability in a concentration- and time-dependent manner (Lin et al., 2015). These flavonoids inhibit PI3K/Akt signaling and reduce Akt phosphorylation at Ser473, thereby releasing FOXO3a from Akt-mediated suppression (Lin et al., 2015). Nuclear FOXO3a then increases transcription of p21 and p27 (Lin et al., 2015). Apigenin can also activate the p53/p21 axis, disrupting cell cycle progression and initiating apoptosis (King et al., 2012; Bao et al., 2022).

Other flavonoids act through related but distinct checkpoints. Quercetin inhibits the proliferation of HepG2 liver cancer cells, although its effects vary among liver cancer cell lines (Jeon et al., 2019). It regulates ROS levels, increases p53 and HO-1 expression, and decreases Cyclin A and CHK1 expression (Jeon et al., 2019). Isorhamnetin activates the p53–p21 axis in pancreatic cancer-associated fibroblasts. It inhibits the Cyclin B1/CDK1 complex and increases CDK1 Tyr15 phosphorylation, leading to G2/M arrest (Ganbold et al., 2025). Naringenin induces G1 arrest in colorectal cancer cells by activating p38-mediated proteasomal degradation of Cyclin D1 (Song et al., 2015). Naringin suppresses β-catenin signaling in triple-negative breast cancer cells, increases p21, decreases survivin, and promotes G1 arrest and apoptosis (Li et al., 2013).

Additional flavonoids converge on the Cyclin/CDK network. Naringenin-loaded nanostructured lipid carriers enhance the effect of oxaliplatin by reducing Cyclin B1 expression and strengthening cell cycle blockade (Raeisi et al., 2019). Neohesperidin inhibits osteosarcoma-cell proliferation and was reported to have limited toxicity toward normal osteoblasts under the tested conditions (Wang S. et al., 2021). It increases p21 and blocks activation of the Cyclin B1/CDK1 complex, thereby inducing G2/M arrest (Wang S. et al., 2021). Fisetin causes G0/G1 arrest in non-small cell lung cancer cells by decreasing Cyclin D1 and CDK4/6 while increasing p21 and p27 (Tabasum and Singh, 2019). In skin cancer cells, fisetin induces apoptosis partly through MTH1 downregulation (Imtiyaz et al., 2023). Phloretin inhibits liver cancer cell proliferation by downregulating Cyclin D1 and inducing G1 arrest (Saraswati et al., 2019). Isoliquiritigenin suppresses proliferation in several tumor types by increasing p21 and p27 and reducing cell cycle proteins such as Cyclin B1 and Cyclin D1 (Zhao et al., 2019; Zeng et al., 2025).

Evidence from fractions enriched in flavonoids provides supportive but lower-specificity evidence for cell cycle regulation. Total flavonoid aglycones from Scutellaria baicalensis show antitumor activity in non-small cell lung cancer models (Wang et al., 2016). High-dose oral treatment reduces xenograft tumor volume, with an inhibitory effect close to that of cisplatin (Wang et al., 2016). In A549 cells, this extract induces S-phase arrest. It suppresses DNA synthesis, decreases Cyclin D1, and increases p53 expression (Wang et al., 2016). These findings support the biological plausibility of flavonoid-class effects on cell cycle regulation, but they are not equivalent to compound-specific pharmacological evidence.

Taken as a group, flavonoids suppress abnormal proliferation by inhibiting upstream proliferative signaling, restoring tumor-suppressive transcriptional programs, and inactivating Cyclin/CDK complexes. The phase of cell cycle arrest, however, varies among tumor types and depends on p53 status, dose, and cell-line background. Cross-cancer validation is therefore needed to identify stable cell cycle targets for therapeutic development.

### 2.2 Induction of multiple forms of regulated cell death

Defective regulated cell death (RCD) is a major mechanism by which tumor cells acquire survival advantages (Liao et al., 2022; Peng et al., 2022). RCD includes apoptosis, autophagy-dependent cell death, ferroptosis, pyroptosis, and other regulated death programs (Liao et al., 2022; Peng et al., 2022). Unlike accidental cell death, RCD is genetically controlled and signal-dependent (Christgen et al., 2022). It is involved in development, tissue homeostasis, and immune responses (Christgen et al., 2022). Tumors frequently disrupt these programs to evade therapy; restoring or redirecting RCD has therefore become an important anticancer strategy (Su et al., 2016; Liao et al., 2022).

Flavonoids can trigger several forms of regulated cell death through overlapping mechanisms (Vetrivel et al., 2019; Nawaz et al., 2025). Apoptosis remains the best-studied form. Naringenin, apigenin, and luteolin decrease anti-apoptotic Bcl-2 and increase pro-apoptotic Bax (Zhang L. et al., 2015; Yan et al., 2018; Raeisi et al., 2019). They also activate caspase-3 and caspase-9, while modulating PI3K/Akt and MAPK signaling (Zhang L. et al., 2015; Yan et al., 2018; Raeisi et al., 2019). Fisetin, puerarin, phloretin, and anthocyanins act mainly on the mitochondrial apoptotic pathway by reducing Bcl-2 and Bcl-xL, increasing Bax and Bad, decreasing mitochondrial membrane potential, and promoting cytochrome c release (Syed et al., 2014b; Saraswati et al., 2019; Chang and Xiao, 2025; Chen et al., 2025). These events activate the caspase-9/caspase-3 cascade (Syed et al., 2014b; Saraswati et al., 2019; Chang and Xiao, 2025; Chen et al., 2025). Isoliquiritigenin also induces mitochondrial apoptosis through Bax upregulation, caspase-3 activation, PARP cleavage, and reduction of Bcl-2, Mcl-1, and survivin (Zhao et al., 2019; Zeng et al., 2025). In HepG2 hepatoma cells, it additionally blocks NF-κB survival signaling via increased cytoplasmic IκBα, diminished nuclear NF-κB activity, and suppressed expression of Bcl-xL and c-IAP1/2, consequently driving caspase-dependent apoptosis (Hsu et al., 2005). In human bladder cancer T24 cells, isoliquiritigenin reduced proliferation and induced concentration-dependent apoptosis, accompanied by loss of mitochondrial membrane potential, upregulation of Bax, Bim, Apaf-1, caspase-9, and caspase-3, and downregulation of Bcl-2 (Si et al., 2017). CDK2 activity increased after treatment, whereas the CDK2 inhibitor MK-8776 attenuated these mitochondrial and apoptotic responses, supporting the involvement of CDK2-associated signaling in this *in vitro* model (Si et al., 2017).

Flavonoids also modulate autophagy-related cell death (Vetrivel et al., 2019; Nawaz et al., 2025). Quercetin, kaempferol, and fisetin can suppress PI3K/Akt/mTOR signaling or activate the AMPK/ULK1 axis (Suh et al., 2010; Aryal et al., 2014; Syed et al., 2014b; Jiang et al., 2015; Deng et al., 2020). These changes affect autophagy-related proteins, including ATG3, ATG5, Beclin1, and LC3 (Suh et al., 2010; Aryal et al., 2014; Syed et al., 2014b; Jiang et al., 2015; Deng et al., 2020). In some tumor models, flavonoids promote LC3-I to LC3-II conversion and drive excessive autophagy-dependent cell death (Suh et al., 2010; Aryal et al., 2014; Syed et al., 2014b; Jiang et al., 2015; Deng et al., 2020). Myricetin induces autophagy in liver cancer cells by downregulating MARCH1, accompanied by an increased LC3-II/LC3-I ratio and reduced P62 expression (Yang et al., 2021).

Ferroptosis is an iron-dependent form of regulated cell death characterized by excessive lipid peroxidation and failure of cellular antioxidant defences (Dixon and Olzmann, 2024; Mishima et al., 2025). Several flavonoids have been linked to ferroptosis in compound- and model-specific studies (Vetrivel et al., 2019; Nawaz et al., 2025). In clear cell renal cell carcinoma, luteolin increased the labile iron pool, depleted glutathione, and promoted lipid peroxidation through excessive HO-1 activity. These effects were partially reversed by deferiprone or ferrostatin-1, supporting an iron- and ferroptosis-dependent component of cell death (Han et al., 2022). In prostate cancer models, luteolin promoted TFEB nuclear translocation and ferritinophagy. TFEB knockdown and pharmacological rescue experiments further supported the involvement of autophagy-dependent ferroptosis (Fu et al., 2024).

Other flavonoids appear to regulate ferroptosis through distinct mechanisms. In breast cancer cells, quercetin promoted TFEB nuclear translocation, lysosomal ferritin degradation, iron release, and lipid peroxidation. TFEB silencing or chloroquine attenuated these effects, supporting a ferritinophagy-dependent mechanism (An and Hu, 2022). Naringenin did not act primarily as an independent ferroptosis inducer at the tested non-toxic concentrations. Instead, it sensitized liver cancer cells to erastin, RSL3, and sorafenib by activating AMPK–PGC-1α signaling and suppressing aerobic glycolysis. Quantitative combination analysis and pharmacological inhibition of AMPK or PGC-1α supported this sensitizing effect (Li et al., 2024).

These findings indicate that flavonoids should not be assigned a uniform ferroptosis mechanism at the class level. Changes in SLC7A11, GPX4, intracellular ROS, or other ferroptosis-associated markers alone are insufficient to establish ferroptotic cell death (Mishima et al., 2025). More convincing evidence requires membrane lipid peroxidation together with functional rescue by established ferroptosis inhibitors, particularly radical-trapping antioxidants such as ferrostatin-1 or liproxstatin-1. Rescue by iron chelators can provide complementary evidence for iron dependence (Chen et al., 2021; Yan et al., 2021; Mishima et al., 2025). Measurements of lipid ROS or lipid-peroxidation products provide additional support, whereas genetic or pharmacological manipulation of key regulators such as GPX4, SLC7A11, ACSL4, or FSP1 can strengthen causal attribution (Dixon and Olzmann, 2024; Mishima et al., 2025). Whenever possible, these approaches should be combined with exclusion of alternative death programs rather than reliance on individual molecular markers (Galluzzi et al., 2018; Dixon and Olzmann, 2024).

Inflammatory forms of cell death have also been reported (Vetrivel et al., 2019; Nawaz et al., 2025). Baicalein and luteolin can activate the NLRP3 inflammasome and promote GSDMD or GSDME cleavage, consistent with pyroptosis-like tumor cell death (Zhou Y.-S. et al., 2022; Liu et al., 2024). Quercetin may regulate the RIPK1/RIPK3/MLKL pathway and induce necroptosis-related responses (Khorsandi et al., 2017). More recent reports link flavonoids to PANoptosis, cuproptosis, or disulfidptosis, although these newer death programs remain less established in flavonoid research and require direct functional validation (Duan et al., 2024; Wendlocha et al., 2024).

A recently described mitochondrial lytic death pathway may broaden the framework for interpreting flavonoid-regulated cell death. Recent studies have suggested that combined innate immune activation and metabolic disruption can induce a caspase-independent lytic death mechanism termed mitoxyperilysis (Wang Y. et al., 2025). In this model, BAX-, BAK1-, and BID-dependent mitochondrial oxidative stress promotes prolonged contact between mitochondria and the plasma membrane. The resulting local oxidative membrane damage, termed mitoxyperiosis, precedes membrane rupture (Wang Y. et al., 2025). Direct evidence linking flavonoids to mitoxyperiosis is not yet available. Nevertheless, flavonoids can affect mitochondrial ROS production, electron transport, cytochrome c, BCL-2-family proteins, mitochondrial ion channels, mitophagy, and mitochondrial dynamics (Lagoa et al., 2011; Testai et al., 2013; Kicinska and Jarmuszkiewicz, 2020; Kicinska et al., 2020; Chang and Xiao, 2025; Zheng X. et al., 2025). Collectively, these actions affect mitochondrial oxidative stress, membrane potential, Ca^2+^ handling, MPTP sensitivity, and mitochondrial quality control. However, these observations provide testable hypotheses rather than evidence that flavonoids induce mitoxyperiosis.

Importantly, the effects of flavonoids on regulated cell death are highly context-dependent rather than uniformly pro-death or cytoprotective (Živanović et al., 2024). A systematic review of plant phenolics found that some compounds could either promote or inhibit ferroptosis depending on the experimental model (Živanović et al., 2024). The main regulatory mechanisms involved the Nrf2/GPX4 and Nrf2/HO-1 pathways and iron metabolism (Živanović et al., 2024). The direction of the response therefore appears to depend not only on the chemical identity of the flavonoid, but also on dose and the pre-existing susceptibility of the cell to oxidative damage. Flavonoids commonly exhibit biphasic or hormetic dose responses, with lower concentrations tending to support antioxidant defence and redox homeostasis, whereas higher concentrations may increase ROS generation and overwhelm cellular antioxidant capacity (Vazhappilly et al., 2025). In ferroptosis, the final outcome may therefore depend on the balance among the Nrf2/GSH/GPX4 antioxidant system, labile iron availability, lipid peroxidation, and iron mobilization. Thus, the same flavonoid may suppress ferroptosis in one setting but promote it in another.

Similar context dependence may also occur in other regulated cell death programs. These pathways should not be viewed as fully independent because ROS accumulation, mitochondrial injury, autophagy, ferroptosis, inflammasome activation, and mitochondria-dependent membrane lysis can overlap within the same cellular context. This complexity also applies to emerging pathways such as mitoxyperiosis and mitoxyperilysis, for which direct evidence involving flavonoids remains limited. Future studies should therefore define the dominant mode and direction of flavonoid-regulated cell death in specific experimental settings. Functional validation should also distinguish primary death mechanisms from secondary stress responses.

### 2.3 Dysregulated oncogenic signaling networks

Oncogenic signaling networks drive tumor growth, survival, invasion, and drug resistance (Zielinska and Katanaev, 2019). These pathways interact with one another and regulate cell cycle progression, metabolic adaptation, immune escape, and resistance to cell death (Zielinska and Katanaev, 2019). NF-κB, ERK, and GPCR-related pathways contribute to inflammatory signaling and proliferative amplification (Sanchez-Vega et al., 2018; Zielinska and Katanaev, 2019). RTK-RAS, PI3K/Akt, p53, and Wnt/β-catenin pathways are also frequently altered through mutation, copy-number variation, or epigenetic dysregulation (Sanchez-Vega et al., 2018; Zielinska and Katanaev, 2019). Altered cell cycle regulators, Myc signaling, and TGF-β signaling further support malignant progression and therapy resistance (Sanchez-Vega et al., 2018). Together, these pathways form a signaling hub that maintains tumor cell survival and proliferation.

Flavonoids can interfere with several components of this oncogenic network (Pandey et al., 2023). In the Wnt/β-catenin pathway, quercetin, genistein, and apigenin can promote β-catenin phosphorylation and degradation (Zhang et al., 2013; Mojsin et al., 2014; Xu et al., 2016). They may also suppress β-catenin nuclear translocation or increase the expression of Wnt antagonists, such as sFRP and DKK (Zhang et al., 2013; Mojsin et al., 2014; Xu et al., 2016). These effects reduce transcription of downstream oncogenic genes, including c-Myc and Cyclin D1 (Zhang et al., 2013; Mojsin et al., 2014; Xu et al., 2016). In the cited models, these changes were associated with reduced proliferation, epithelial–mesenchymal transition, and stemness-related phenotypes.

The PI3K/Akt pathway is another signaling pathway frequently affected by flavonoids (Pandey et al., 2023). Kaempferol, baicalein, and fisetin can inhibit PI3K activity and reduce Akt phosphorylation at Ser473 or Thr308 (Chin et al., 2018; Li Q. et al., 2019; Kubina et al., 2022). This decreases downstream mTOR signaling and limits tumor growth, metabolic adaptation, and survival signaling (Chin et al., 2018; Li Q. et al., 2019; Kubina et al., 2022). Puerarin also reduces PI3K and Akt phosphorylation in a dose-dependent manner (Chen et al., 2025). This effect can be reversed by the PI3K activator 740Y-P, supporting the involvement of this pathway (Chen et al., 2025).

Several flavonoids also suppress NF-κB signaling (Pandey et al., 2023). Apigenin, EGCG, luteolin, and fisetin inhibit the IKKα/β complex and prevent IκBα phosphorylation and degradation (Shukla et al., 2007; Johnson et al., 2015; Shukla et al., 2015; Kubina et al., 2022; Suhail et al., 2022). This blocks NF-κB p65 nuclear translocation (Shukla et al., 2007; Johnson et al., 2015; Shukla et al., 2015; Kubina et al., 2022; Suhail et al., 2022). It also reduces the expression of TNF-α, IL-6, MMP-2, and MMP-9 (Shukla et al., 2007; Johnson et al., 2015; Shukla et al., 2015; Kubina et al., 2022; Suhail et al., 2022). Through this mechanism, flavonoids may limit inflammatory signaling, invasion, and metastatic potential (Shukla et al., 2007; Johnson et al., 2015; Shukla et al., 2015; Kubina et al., 2022; Suhail et al., 2022).

The MAPK/ERK pathway is also frequently affected by flavonoids (Pandey et al., 2023). Genistein and kaempferol inhibit ERK1/2 phosphorylation (Lee and Kim, 2016; Shin et al., 2017). Fisetin acts upstream by inhibiting Raf-1 kinase activity, thereby suppressing the Ras–Raf–MEK–ERK cascade and reducing tumor-cell proliferation and migration (Kubina et al., 2022). In melanoma models, fisetin also directly binds to and inhibits mTOR and p70S6K. This activity was associated with reduced AKT, mTOR, and p70S6K phosphorylation and inhibition of melanoma progression in conventional cell cultures, three-dimensional human melanoma skin equivalents, and xenograft models (Syed et al., 2014a). Anthocyanins can increase KLF4 expression and then reduce ERK and p38 phosphorylation (Chang and Xiao, 2025).

STAT3-related signaling is also affected by several flavonoids (Pandey et al., 2023). Phloretin binds to the N-SH2 domain of SHP-1 and disrupts its autoinhibited conformation (Saraswati et al., 2019). This increases SHP-1 phosphatase activity and suppresses STAT3 Tyr705 phosphorylation and nuclear translocation (Saraswati et al., 2019). It also reduces phosphorylation of JAK2, AKT, mTOR, ERK, and VEGFR2, which suggests a broader effect on proliferative signaling (Saraswati et al., 2019). Isoliquiritigenin directly reduces STAT3 Tyr705 phosphorylation and nuclear translocation. It also inhibits JAK2 activity, thereby blocking the JAK2/STAT3 cascade (Zhao et al., 2019).

Although these pathways differ in molecular composition, they converge on shared effectors such as c-Myc, Cyclin D1, MMPs, inflammatory cytokines, and anti-apoptotic proteins. Flavonoids therefore appear to function as signaling-network modulators rather than single-target inhibitors. Most studies, however, still validate isolated pathways in single-cell models. Rescue experiments, phosphoproteomics, and network-based analyses are needed to define causal hierarchy and compensatory feedback.

### 2.4 Epigenetic regulation and non-coding RNA networks

Epigenetic dysregulation is a key feature of cancer (Ilango et al., 2020). It includes abnormal DNA methylation, altered histone modifications, and disordered non-coding RNA expression (Ilango et al., 2020). Promoter CpG island hypermethylation can silence tumor suppressor genes such as MLH1 and p16INK4a, which are frequently involved in colorectal cancer, lung cancer, and other malignancies (Baylin and Jones, 2011; Ilango et al., 2020). Histone modification also shapes gene expression; excessive HDAC activity can compact chromatin and silence tumor-suppressive genes (Dong et al., 2012).

Non-coding RNAs add another layer to this regulatory system. Our previous work showed that non-coding RNAs can act as core components of epigenetic regulation in cancer (Wang J. et al., 2025). Tumor-suppressive miRNAs, such as miR-34a and miR-137, may be silenced by promoter methylation. In contrast, oncogenic miRNAs, such as miR-9, can repress tumor suppressor genes (Lodygin et al., 2008; Sato et al., 2011). Long non-coding RNAs and circular RNAs also regulate chromatin structure, RNA stability, and signaling output (Lodygin et al., 2008; Sato et al., 2011). Together, these RNA-mediated processes contribute to tumor progression and therapy resistance (Wang J. et al., 2025).

Flavonoids can reshape this network through several mechanisms (Jiang et al., 2021). Genistein downregulates the oncogenic miR-1260b. This releases the Wnt pathway inhibitors sFRP1 and Dkk2 from miRNA-mediated suppression (Hirata et al., 2013). It can also induce demethylation of the Wnt5a promoter, thereby reducing malignant behavior (Hirata et al., 2013). EGCG regulates lncRNA expression and affects histone-modifying enzyme activity, which suppresses gastric cancer cell proliferation and migration (Zhao et al., 2020). Naringenin and naringin may influence gene expression through non-coding RNA-associated epigenetic mechanisms, including the regulation of miRNAs, lncRNAs, and circRNAs (Wu et al., 2025). Naringenin enantiomers have been reported to modulate miR-17-3p expression in an oxidative-stress model, although the relevance of this finding to cancer remains uncertain (Curti et al., 2017). Luteolin inhibits DNMT and HDAC activity, which promotes the re-expression of tumor suppressor genes (Selvi et al., 2015; Zuo et al., 2018). However, its effects on specific circRNA–miRNA–mRNA networks in cancer require further validation.

Epigenetic and non-coding RNA regulation provides a higher-level explanation for the multi-target effects of flavonoids. DNMTs, HDACs, miRNAs, lncRNAs, and circRNAs can influence multiple downstream processes, including Wnt/β-catenin signaling, PI3K/Akt signaling, EMT, apoptosis, and drug tolerance. Changes in these regulatory networks may therefore connect flavonoid exposure with several malignant phenotypes. However, these observations do not necessarily indicate that epigenetic regulators are the primary molecular targets of flavonoids. Their contribution appears to vary with cell type, treatment conditions, and the genetic and metabolic state of the tumor.

An important issue is whether flavonoid-associated epigenetic alterations represent primary pharmacological effects or secondary responses to cellular stress. For some flavonoids, stronger evidence for a primary epigenetic mechanism comes from studies showing changes in DNMT or HDAC enzymatic activity together with promoter remodeling and gene re-expression (Fang et al., 2005; Sundaram et al., 2018; Kedhari Sundaram et al., 2019). However, even functional involvement of an epigenetic pathway does not necessarily demonstrate direct molecular targeting. In contrast, changes in DNMT or HDAC expression, histone marks, DNA methylation, or non-coding RNA profiles observed under substantial oxidative stress may reflect downstream stress-associated responses rather than primary epigenetic effects (García-Guede et al., 2020). Cellular oxidative stress itself can alter DNA methylation, histone modifications, and non-coding RNA regulation, making temporal sequence important when interpreting these epigenetic changes (García-Guede et al., 2020). Such findings should therefore be interpreted cautiously. Time-course studies, direct enzyme or target-engagement assays, and genetic or pharmacological rescue experiments are needed to distinguish primary epigenetic reprogramming from downstream stress-associated changes.

### 2.5 Metabolic reprogramming

Metabolic reprogramming is a core feature of malignant tumors (Faubert et al., 2020). Cancer cells remodel nutrient use and energy production to support growth, invasion, and survival under microenvironmental stress (Faubert et al., 2020). This adaptation extends beyond glycolysis to glutamine metabolism, fatty acid metabolism, and other nutrient networks (Vander Heiden et al., 2009; Kim and DeBerardinis, 2019; Pavlova et al., 2022). It is shaped by oncogenic signaling as well as external pressures such as hypoxia and nutrient deprivation (Kamphorst et al., 2015; Samec et al., 2021). Flavonoids may affect tumor metabolism by targeting both upstream signaling pathways and downstream metabolic enzymes.

HIF-1 is a central regulator of tumor metabolic adaptation under hypoxia (Samec et al., 2021). Several flavonoids can interfere with HIF-1 activity and its downstream metabolic targets (Melstrom et al., 2011; Wu et al., 2019). These targets include glucose transporters, hexokinase II, and lactate dehydrogenase A (Melstrom et al., 2011; Wu et al., 2019). Naringin inhibits HIF1A transcriptional activity in colon cancer cells (Pan et al., 2023). It also downregulates ENO2, which reduces aerobic glycolysis and suppresses cell proliferation and invasion (Pan et al., 2023). In A375 melanoma cells, naringin decreases HIF-1α, PKM2, and LDHA expression, thereby weakening the Warburg effect (Guo et al., 2016).

Kaempferol regulates HIF-1 through a different mechanism (Mylonis et al., 2010). Under hypoxic conditions, it strongly suppresses HIF-1 activity in a dose-dependent manner (Mylonis et al., 2010). This effect does not mainly result from reduced HIF-1α protein expression (Mylonis et al., 2010). Instead, kaempferol inhibits p44/42 MAPK phosphorylation and blocks HIF-1α nuclear localization, leaving HIF-1α in the cytoplasm and reducing its transcriptional activity (Mylonis et al., 2010).

Quercetin also affects metabolic reprogramming through PI3K/Akt/mTOR, Wnt/β-catenin, and MAPK/ERK1/2 signaling (Granato et al., 2017; Erdogan et al., 2018; Jia et al., 2018). It reduces the expression or activity of glycolytic regulators such as GLUT1, PKM2, and LDH-A, thereby lowering glucose uptake and lactate production (Granato et al., 2017; Erdogan et al., 2018; Jia et al., 2018). Quercetin can also interfere with monocarboxylate transporter-mediated lactate export, which may weaken the acidic microenvironment that favors tumor cell survival (Vera et al., 2001; Maurya and Vinayak, 2015; Jia et al., 2018). In AGS gastric cancer cells, baicalein acts through the PI3K/Akt/HIF-1α axis; inhibition of Akt phosphorylation reduces HKII, PDK1, and LDHA expression (Chen et al., 2015).

Current evidence suggests that HIF-1α, PI3K/Akt/mTOR, Wnt/β-catenin, and MAPK/ERK are not isolated metabolic regulators. They form an upstream network that supports tumor metabolic adaptation. Flavonoids may modulate components of this network and reduce the expression of GLUT1, HKII, PKM2, LDHA, ENO2, and MCTs. The resulting metabolic shift may decrease glucose uptake, lactate production, and adaptation to acidic stress.

Metabolic phenotypes, however, are highly sensitive to experimental context. Oxygen tension, nutrient supply, cell density, and assay design can all influence the results. Reduced expression of selected glycolytic enzymes is not sufficient to prove reversal of metabolic reprogramming. Metabolic flux analysis, isotope tracing, and *in vivo* metabolic imaging are needed to define the extent to which flavonoids reshape tumor metabolism. The integrated tumor cell-intrinsic mechanisms discussed above are summarized in Figure 2.

**FIGURE 2:**
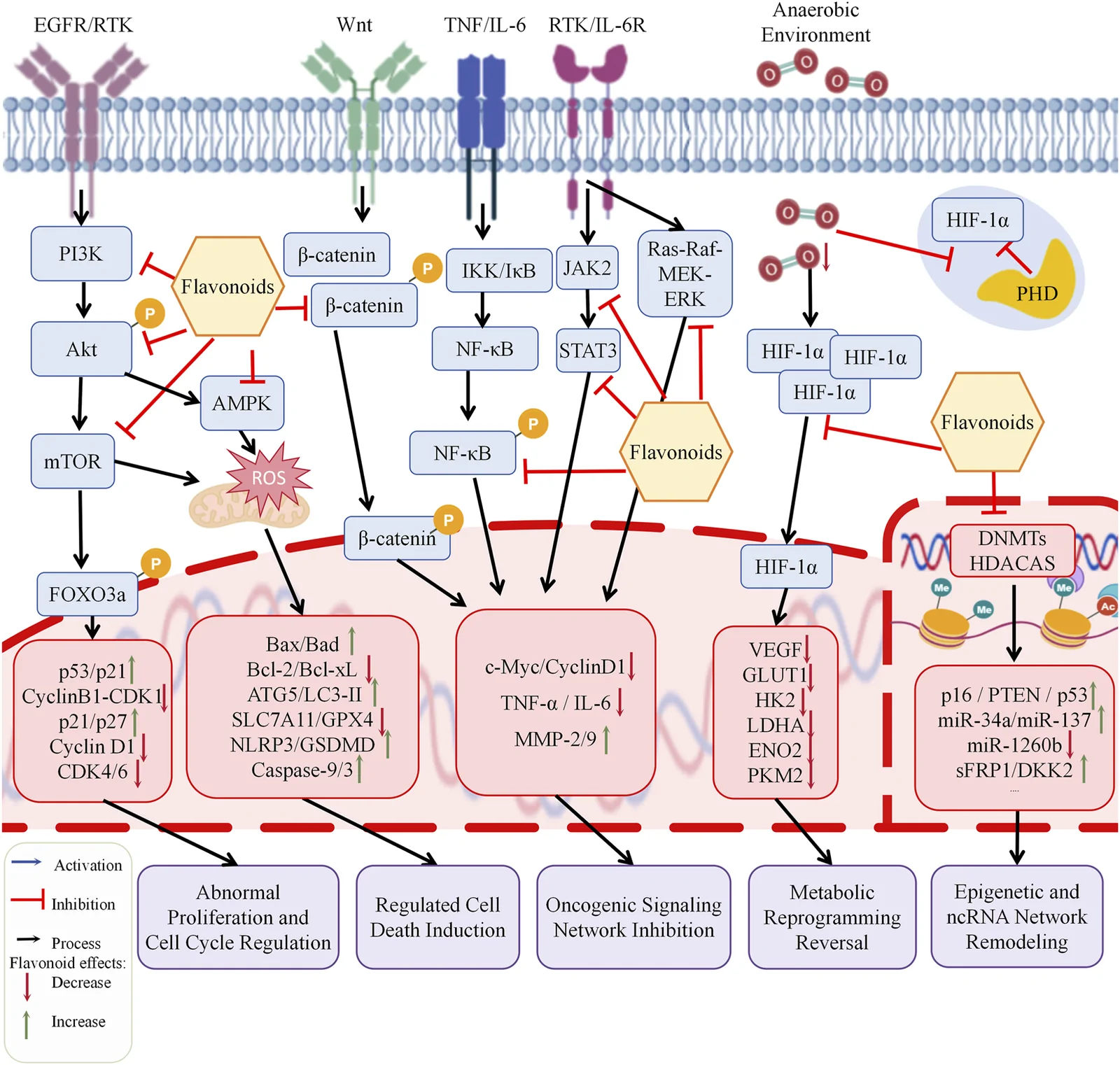
Multi-target signaling mechanisms by which flavonoids regulate tumor cell-intrinsic cancer hallmarks.

## 3 Interactions between flavonoids and the tumor microenvironment

### 3.1 Inflammatory signaling and mitochondria

Chronic low-grade inflammation is a core feature of the TME (Yu et al., 2022). It promotes tumor initiation and progression and also contributes to the failure of chemotherapy, targeted therapy, and immunotherapy (Yu et al., 2022). Within the TME, NF-κB and STAT3 are not limited to tumor cell-intrinsic signaling. They also mediate inflammatory crosstalk among tumor cells, CAFs, tumor-associated macrophages (TAMs), and immunosuppressive cells (Nishida and Andoh, 2025).

Persistent activation of these pathways increases the secretion of TGF-β, IL-6, and TNF-α (Nishida and Andoh, 2025). These cytokines promote CAF activation and recruit Tregs and M2-like macrophages, forming a physical and immunosuppressive barrier that limits drug penetration and weakens immune-cell attack (Zheng L. et al., 2025). Mitochondrial damage-associated molecular patterns can further activate innate immune pathways, including RIG-I and cGAS. This amplifies inflammatory signaling in the TME (Xia et al., 2025).

Flavonoids can modulate these inflammatory circuits while affecting tumor cell metabolism and mitochondrial integrity (Lim et al., 2019; Koklesova et al., 2021). Quercetin inhibits PI3K/Akt/mTOR and MAPK/ERK signaling, promotes apoptosis, and reduces tumor cell proliferation (Silva-Pinto et al., 2025). It also suppresses NF-κB activity and regulates JAK/STAT signaling (Silva-Pinto et al., 2025). Together, these findings suggest that quercetin can alter inflammatory and tumor cell signaling, although its effects on immune recognition require direct functional validation.

Genistein decreases mitochondrial membrane potential and promotes cytochrome c release, leading to intrinsic apoptosis (de Oliveira, 2016). In A549 and 95D lung cancer cells, genistein increases mitochondrial ROS production and damages mitochondrial proteins and mtDNA (Chan et al., 2022). These changes increase the sensitivity of tumor cells to apoptosis. Genistein may also alter oxidative phosphorylation enzyme activity and ATP generation (de Oliveira, 2016).

Fisetin acts on both inflammatory and mitochondrial pathways. It inhibits NF-κB and JAK/STAT3 signaling and reduces pro-inflammatory cytokine secretion in the TME (Kubina et al., 2022). It also increases mitochondrial ROS production, which leads to mtDNA damage, respiratory chain dysfunction, and mitochondria-dependent apoptosis (Syed et al., 2014b).

Inflammation connects tumor progression, immune escape, and therapy tolerance. Sustained NF-κB and STAT3 activation can recruit immunosuppressive cells and induce EMT-related transcription factors and resistance-associated genes (Yu et al., 2022; Nishida and Andoh, 2025). This reciprocal relationship among inflammation, immunosuppression, and treatment tolerance forms a protumor network. Flavonoids may disrupt parts of this network by suppressing inflammatory signaling and altering the immune contexture, but these effects require compound- and model-specific validation.

### 3.2 Immune regulation and reversal of immune escape

Metabolic reprogramming in the TME is closely linked to immune escape (Arner and Rathmell, 2023). This process is not driven by tumor cells alone; it emerges from interactions among tumor cells, immune cells, and stromal components (De Martino et al., 2024; Lyu et al., 2025). Tumor cells compete with effector T cells for glucose and other nutrients (Chang et al., 2015). Tumor-derived metabolites, including lactic acid, kynurenine, and adenosine, inhibit cytotoxic T cells and natural killer cells and promote Treg expansion and M2-like TAM polarization (Brand et al., 2016; Watson et al., 2021).

Targeting immunometabolic crosstalk is therefore an important strategy for restoring antitumor immunity (Scharping et al., 2016). Such intervention may reduce T-cell exhaustion, weaken immunosuppressive cell function, and improve the metabolic fitness of immune cells used in adoptive cell therapy (Scharping et al., 2016). Flavonoids may contribute to this process by regulating immune metabolism and suppressing immune escape (Yan and Wang, 2025).

Flavonoids have been reported to shift tumor-associated macrophage polarization from M2-like toward M1-like states in selected models (Yan and Wang, 2025). Luteolin can limit the infiltration of macrophages and monocytes into the TME by reducing CCL2 secretion from TAMs (Choi et al., 2016). Isoliquiritigenin suppresses M2-like polarization through PGE2/PPAR-δ and IL-6/STAT3 signaling (Zhao et al., 2014). Baicalein acts on the PI3Kγ/NF-κB axis, reduces IL-10 and TGF-β expression, suppresses M2 polarization, and promotes M1 polarization (He et al., 2021).

Some flavonoids can modulate Tregs and other immunosuppressive cell populations, suggesting a role in relieving immune suppression within the TME (Chen et al., 2022). Wogonin targets the TGF-β1 signaling axis. It inhibits Smad3, GSK-3β, and ERK1/2 activity, while enhancing p38 MAPK phosphorylation (Dandawate et al., 2012). Through these effects, it regulates Treg differentiation and function (Dandawate et al., 2012). Naringenin inhibits protein kinase C activity and blocks TGF-β1 transport, thereby reducing extracellular TGF-β1 levels both within the TME and systemically (Zhang et al., 2016). As a result, Tregs and myeloid-derived suppressor cells (MDSCs) are reduced. CD8^+^ T-cell activation and IFN-γ production are increased (Zhang et al., 2016). Scutellarin disrupts the interaction between TNF and TNFR2 (Chen et al., 2022). This blocks downstream p38 MAPK activation, suppresses TNFR2^+^ Treg expansion, and promotes IFN-γ-producing CD8^+^ T-cell infiltration (Chen et al., 2022).

Flavonoids may also attenuate tumor immune escape by regulating immune checkpoint molecules, particularly PD-L1 (Ke et al., 2019; Yan and Wang, 2025). Baicalein and baicalin suppress STAT3 activity and IFN-γ-induced PD-L1 expression, which may increase tumor cell susceptibility to T-cell-mediated responses (Ke et al., 2019). Apigenin combined with curcumin reduces STAT1 phosphorylation and inhibits IFN-γ-induced PD-L1 upregulation in melanoma cells (Xu et al., 2018). Hesperidin downregulates PD-L1 in breast cancer cells by inhibiting AKT and NF-κB signaling (Kongtawelert et al., 2020). Icaritin binds to IKK-α and prevents activation of the IKK complex, thereby disrupting NF-κB signaling and reducing PD-L1 expression (Mo et al., 2021). Isoliquiritigenin inhibits STAT3 phosphorylation and decreases PD-L1 expression on tumor cells, thereby limiting PD-1/PD-L1-associated immune escape in the tested models (Zeng et al., 2025).

In summary, preclinical studies suggest that flavonoids may modulate selected immune-cell programs and checkpoint-associated markers. The strength of inference varies substantially across tumor cell assays, coculture systems, and *in vivo* models. Combination with immunotherapy therefore remains a hypothesis requiring compound-specific exposure, immune-function assays, and validation in clinically relevant models.

### 3.3 Angiogenesis and the hypoxic microenvironment

Hypoxia is common in solid tumors and occurs when oxygen demand exceeds the supply from existing blood vessels (Schito, 2018). Under hypoxia, HIF-1α induces genes involved in angiogenesis, invasion, metabolic adaptation, EMT, and immune escape (Schito, 2018; Jiang et al., 2020). Because tumor vessels are often abnormal and poorly functional, they fail to fully relieve hypoxia and instead sustain a hypoxia–angiogenesis feedback loop (Schito, 2018; Jiang et al., 2020).

Hypoxia also promotes HIF-1α-dependent vasculogenic mimicry, in which tumor cells form vessel-like channels to support oxygen and nutrient supply (Wang et al., 2017; Jiang et al., 2020). Beyond its role in tumor metabolic reprogramming discussed in Section 2.5, HIF-1α also drives angiogenesis and hypoxia-related feedback within the TME.

Several flavonoids regulate HIF-1α-associated signaling in experimental models. Apigenin reduces HIF-1α and VEGF expression in ovarian cancer cells and suppresses angiogenesis in chorioallantoic membrane and Matrigel plug assays (Fang et al., 2007). Apigenin and isoliquiritigenin also inhibit endothelial-cell proliferation, migration, and tube formation and reduce tumor microvessel density (Fu et al., 2022; Zeng et al., 2025). These effects involve suppression of HIF-1α and the VEGF-A/VEGFR2 axis (Fu et al., 2022; Zeng et al., 2025).

In a cobalt chloride-induced hypoxia model, luteolin downregulated angiogenic factors, inhibited hypoxia-associated angiogenesis, and attenuated STAT3 signaling (Fang et al., 2018). Luteolin also suppresses the proangiogenic activity of M2-like TAMs by downregulating HIF-1α-related signaling, thereby reducing angiogenesis and invasive behavior in the tested models (Fang et al., 2018).

In multiple myeloma models, Wogonin enhances prolyl hydroxylation and VHL-dependent ubiquitination of HIF-1α (Fu et al., 2016). It also inhibits Hsp90 binding. These effects reduce HIF-1α protein stability and decrease secretion of VEGF, PDGF, and bFGF (Fu et al., 2016). These coordinated tumor-microenvironment effects are summarized in Figure 3.

**FIGURE 3:**
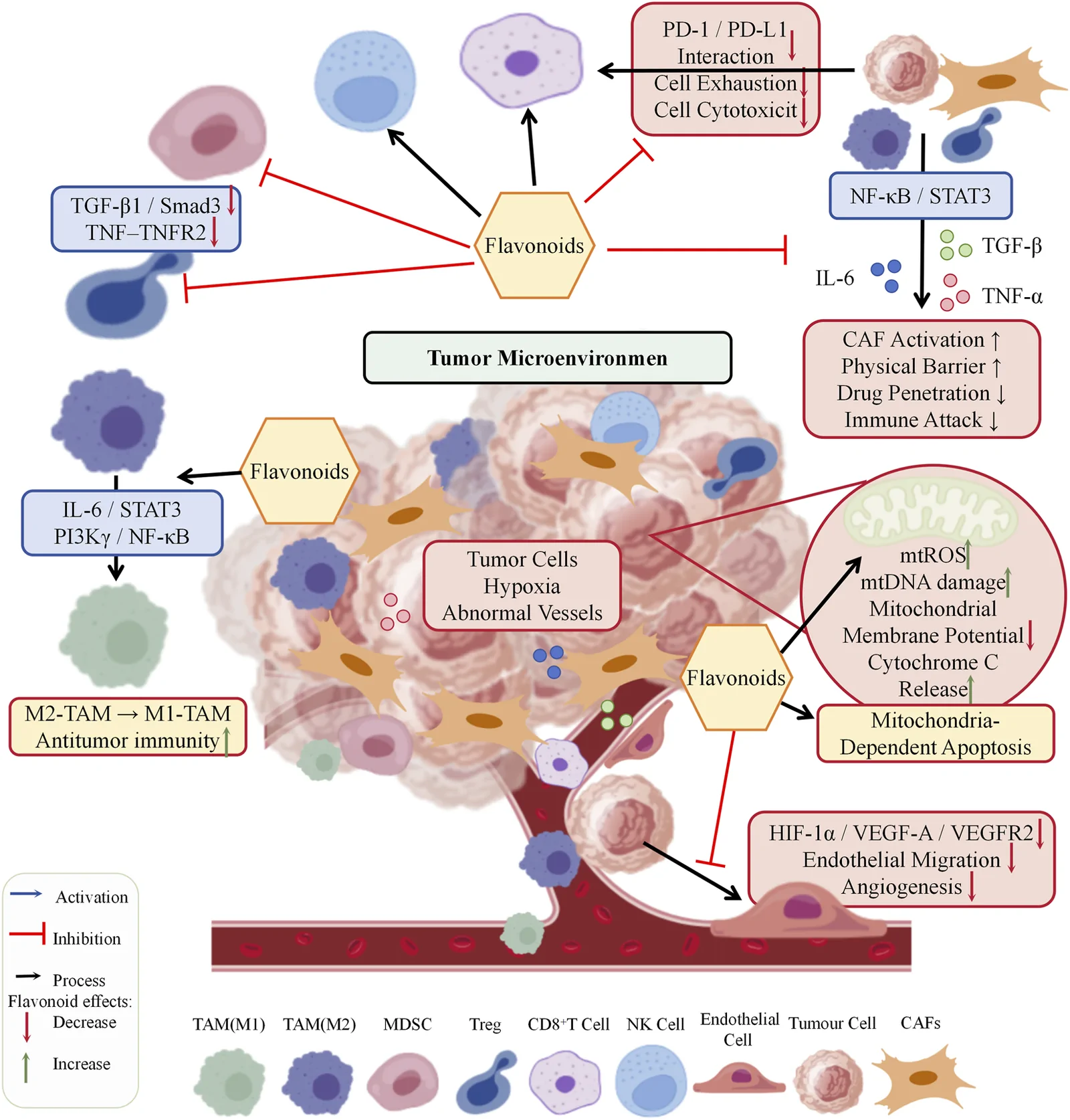
Flavonoid-mediated remodeling of the tumor microenvironment through inflammatory, immune, and angiogenic signaling networks.

## 4 The role of flavonoids in cancer drug resistance

### 4.1 Reversal of multidrug resistance and drug efflux

Multidrug resistance (MDR) is a major cause of chemotherapy failure in cancer (Wang J. Q. et al., 2021). A central mechanism of MDR is the abnormal activation of ATP-binding cassette (ABC) transporters (Wang J. Q. et al., 2021). Among them, P-glycoprotein (P-gp/ABCB1) and multidrug resistance-associated proteins (MRPs/ABCC family) are the best characterized (Wang J. Q. et al., 2021). These transporters use ATP hydrolysis to export structurally diverse anticancer drugs, including doxorubicin, paclitaxel, and methotrexate (Schinkel and Jonker, 2003).

MRPs also transport endogenous organic anions, such as leukotriene C4, bile acids, and cyclic nucleotides (Zelcer et al., 2003). Their overexpression can reduce intracellular drug accumulation and weaken cytotoxic efficacy (Bansal et al., 2009). The MDR phenotype is further reinforced by increased drug metabolism, mutations in drug targets, enhanced DNA repair, and high expression of anti-apoptotic proteins (Lohner et al., 2007). Together, these changes allow tumor cells to tolerate multiple chemotherapeutic and targeted agents.

Flavonoids can reverse MDR mainly by modulating the function or expression of ABC transporters, particularly P-gp, a key mediator of MDR (Ye et al., 2019; Dong J. et al., 2023). Quercetin, EGCG, baicalein, apigenin, luteolin, kaempferol, nobiletin, and tangeretin have all been reported to inhibit P-gp activity (Bai et al., 2019; Fang et al., 2021). Quercetin, apigenin, and luteolin can competitively bind to the substrate-binding pocket of P-gp (Bai et al., 2019; Fang et al., 2021). This blocks the interaction between P-gp and drugs such as doxorubicin or paclitaxel, thereby reducing drug efflux (Bai et al., 2019; Fang et al., 2021).

Some flavonoids also interfere with the energy supply required for P-gp transport. Quercetin, EGCG, kaempferol, and nobiletin can bind to the nucleotide-binding domains of P-gp and suppress ATP hydrolysis (Wang et al., 2005; Fang et al., 2021). This disruption of the efflux cycle increases intracellular drug retention. Quercetin, baicalein, and silibinin can additionally reduce P-gp protein production by downregulating MDR1 transcription or translation (Limtrakul et al., 2005; Bhutto et al., 2018).

Structure–activity relationship studies suggest that P-gp inhibition depends on specific flavonoid structures (Conseil et al., 1998; Sheu et al., 2010; Fang et al., 2021). The 5-hydroxyl group on ring A, the 3-hydroxyl group on ring C, and the C2 = C3 double bond are important for inhibitory activity (Conseil et al., 1998; Sheu et al., 2010; Fang et al., 2021). Flavonols such as quercetin and kaempferol, as well as chalcones, show stronger P-gp binding than flavanones or isoflavones. This may be related to their planar structure and hydrophobic properties (Conseil et al., 1998; Sheu et al., 2010; Fang et al., 2021).

Flavonoids also inhibit members of the MRP family, including MRP1 and MRP2 (Fang et al., 2019; Gonçalves et al., 2020; Padder, 2025). Their effects often differ depending on the subtype. Molecular-docking analyses suggest that rutin, taxifolin, and myricetin can interact with residues in nucleotide-binding domain 1 of MRP1, including SER686 and SER689 (Padder, 2025). These computational findings do not establish transporter inhibition or multidrug-resistance reversal. Experimental assays of transporter activity and intracellular drug accumulation are required. Flavonoid dimer derivatives containing triazole groups, such as compound 78, can also bind competitively to the bipartite binding site of MRP1 (Wong et al., 2018; Gonçalves et al., 2020). This interaction has been associated with disrupted substrate recognition and reversal of MRP1-mediated multidrug resistance in the reported models (Wong et al., 2018; Gonçalves et al., 2020).

For MRP2, baicalein, tangeretin, kaempferol, and silibinin may act as competitive substrates. They bind to the substrate-binding pocket and inhibit MRP2-mediated drug efflux (Fang et al., 2019). This can increase intracellular drug concentration and improve cytotoxic effects (Fang et al., 2019). Structure–activity analyses indicate that 3-OH, 5-OH, 6-OH, and 4′-OCH_3_ substitutions contribute to MRP2 binding affinity. MK-571, a specific MRP2 inhibitor, can further enhance flavonoid uptake and strengthen transporter inhibition (Fang et al., 2019).

Selectivity remains a critical issue. Some flavonoids may show partial substrate selectivity toward MRP2 *in vitro* (Gonçalves et al., 2020), but this does not establish physiological safety (Li and Paxton, 2013). ABC transporters involved in tumor MDR also serve important functions in normal tissues (Li and Paxton, 2013; Taskar et al., 2022). P-gp and BCRP limit intestinal absorption and restrict the brain penetration of many xenobiotics, whereas MRP2 contributes to biliary and renal excretion and transports endogenous conjugates (Järvinen et al., 2021; Taskar et al., 2022). Inhibition of these transporters may therefore alter the absorption, distribution, and elimination of co-administered drugs rather than acting only within tumors.

Intestinal P-gp or BCRP inhibition can increase systemic exposure to substrate drugs, whereas sufficiently strong inhibition at the blood–brain barrier may increase CNS exposure (Kalvass et al., 2013; Taskar et al., 2022). Interference with hepatic or renal efflux may also modify drug clearance, although the magnitude of this effect depends on the transporter, substrate, and site of inhibition (Taskar et al., 2022). Clinical evidence supports the possibility of such interactions. In healthy volunteers, repeated quercetin administration increased the AUC and Cmax of the P-gp substrate fexofenadine by approximately 55% and 68%, respectively (Kim et al., 2009). These pharmacokinetic changes may enhance antitumor drug exposure but can also increase off-target toxicity and drug–drug interaction risks, particularly for agents with narrow therapeutic windows. Therefore, transporter inhibition observed in cultured tumor cells should not be interpreted as tumor-selective reversal of MDR. Potential systemic safety risks and pharmacokinetic consequences of ABC-transporter inhibition in normal tissues also require consideration (Li and Paxton, 2013; Taskar et al., 2022).

### 4.2 Intervention in epithelial–mesenchymal transition

Epithelial–mesenchymal transition (EMT) is a key driver of tumor progression, promoting invasion, metastasis, and treatment resistance (Zhang and Weinberg, 2018; Proença et al., 2023; Mohammad et al., 2025). EMT involves the loss of epithelial markers and acquisition of mesenchymal features, thereby increasing tumor cell migration and invasion (Yang et al., 2020; Xie et al., 2025). During this process, tumor cells also gain stem cell-like traits and resistance to apoptosis (Fischer et al., 2015; Shibue and Weinberg, 2017; Mohammad et al., 2025).

Flavonoids can inhibit EMT by regulating several EMT-related pathways (Proença et al., 2023; Mohammad et al., 2025). One major target is the TGF-β/Smad pathway. Anthocyanins suppress TGF-β-induced Smad2 phosphorylation and reduce nuclear translocation of the Smad2/3–Smad4 complex (Ouanouki et al., 2017). This increases E-cadherin expression and decreases Snail and fibronectin levels, thereby reversing the mesenchymal phenotype of glioma cells (Ouanouki et al., 2017). 3,6-Dihydroxyflavone inhibits TGF-β-induced EMT and migration in breast epithelial cells. This effect is associated with suppression of Notch-related signaling and reduced EMT marker remodeling (Chen et al., 2016).

The Wnt/β-catenin pathway is another important EMT-related target of flavonoids (Yu et al., 2021). Baicalein downregulates Wnt1 expression and reduces β-catenin signaling (Ma et al., 2016). This decreases the expression of downstream EMT-related genes and suppresses EMT and lung metastasis in breast cancer models (Ma et al., 2016).

NF-κB and STAT3 signaling also promote EMT by activating transcription factors (Huang et al., 2022). Luteolin inhibits IκB phosphorylation and NF-κB p65 nuclear translocation, thereby reducing the transcriptional activation of Snail and Twist (Ruan et al., 2012). It also decreases integrin β1 and focal adhesion kinase expression, thereby reversing hypoxia-induced EMT in lung cancer cells (Ruan et al., 2012). Fisetin and isoliquiritigenin suppress NF-κB and STAT3 signaling and downregulate Snail, Twist, and ZEB1 while increasing E-cadherin and reducing N-cadherin, vimentin, and fibronectin (Tabasum and Singh, 2019; Zeng et al., 2025).

These changes restore epithelial features and limit invasive behavior. Fisetin and isoliquiritigenin also reduce MMP-2 and MMP-9 secretion and activity (Tabasum and Singh, 2019; Zeng et al., 2025). This decreases extracellular matrix degradation and further suppresses invasion (Tabasum and Singh, 2019; Zeng et al., 2025). In addition, fisetin lowers the expression of stemness markers, including CD44, CD133, and ALDH1 (Tabasum and Singh, 2019). This may reduce cancer stem cell-like properties and limit the emergence of metastatic and drug-resistant clones (Tabasum and Singh, 2019). In Caco-2 cells, puerarin increases E-cadherin and decreases vimentin. This reduces migration and invasion. The effect depends, at least partly, on inhibition of the PI3K/Akt pathway (Chen et al., 2025).

EMT is closely connected to cancer stem cell-like phenotypes (Shibue and Weinberg, 2017). EMT-associated transcription factors can increase the expression of CD44, CD133, ALDH1, and other stemness markers (Shibue and Weinberg, 2017). Some flavonoids, such as EGCG and genistein, may reduce stemness while suppressing EMT (Naujokat and McKee, 2021; Yang X. et al., 2022). This dual effect may help limit both metastasis and therapy resistance.

Taken together, flavonoids may attenuate EMT-associated resistance through three broad mechanisms: suppression of EMT-driving pathways, restoration of epithelial markers with reduction of mesenchymal markers, and inhibition of stemness-associated and invasive phenotypes. These preclinical findings provide a rationale for testing selected flavonoids in combination strategies for metastatic or drug-resistant tumors. Their relevance, however, depends on compound exposure, tumor context, and functional validation in resistant models.

### 4.3 Epigenetic alterations and resistance reversal

Epigenetic dysregulation contributes to drug resistance by altering gene expression without changing the DNA sequence (Hogg et al., 2020; Pang et al., 2024). It includes abnormal DNA methylation, histone modification, and RNA-based regulation (Hogg et al., 2020; Pang et al., 2024). These changes can silence tumor suppressor genes, activate resistance-related pathways, and reshape the immune microenvironment (Izzo et al., 2020; Selvakumar et al., 2020; To and Cho, 2021). As a result, tumor cells acquire stronger drug efflux capacity, EMT activity, anti-apoptotic signaling, and therapeutic tolerance (Izzo et al., 2020; Selvakumar et al., 2020; To and Cho, 2021). Flavonoids may restore more favorable gene expression patterns by targeting DNA methylation, histone modification, and non-coding RNA networks (Izzo et al., 2020; To and Cho, 2021).

EGCG targets DNA methylation by competitively binding the catalytic domain of DNMT1 and inducing promoter demethylation of tumor suppressor genes such as SCUBE2, p16, and WIF-1 (Gao et al., 2009; Meng et al., 2017; Sheng et al., 2019). Whether these changes functionally reverse drug resistance requires validation in matched resistant models. Quercetin inhibits DNMT1 and DNMT3A/B activity in a dose-dependent manner. In cervical cancer cells, it reduces aberrant methylation of genes including CDH1 and TIMP3 and increases cisplatin sensitivity (Kedhari Sundaram et al., 2019).

Genistein has been reported to modify DNA methylation and chromatin-associated regulation of tumor-suppressive genes, including BRCA1/2, ERβ, p21, and p16, in breast or prostate cancer models (Majid et al., 2008; Bosviel et al., 2012; Mahmoud et al., 2015). In prostate cancer cells, genistein upregulated miR-34a and suppressed the oncogenic lncRNA HOTAIR, contributing to inhibition of cell growth (Chiyomaru et al., 2013). More recently, integrated network, single-cell, and spatial transcriptomic analyses identified HSD17B1 as a potential genistein-associated target in gastric adenocarcinoma (Wang X. et al., 2025). These studies support model-dependent RNA and transcriptional effects of genistein but do not establish restoration of endocrine therapy sensitivity.

At the level of histone modification, apigenin inhibits the expression and activity of HDAC1 and HDAC3 (Pandey et al., 2012; Tseng et al., 2017). This increases histone H3 and H4 acetylation and upregulates genes associated with cell cycle arrest and apoptosis, including p21 and Bax (Pandey et al., 2012; Tseng et al., 2017). These effects may contribute to reversal of chemoresistance in prostate and breast cancer models (Pandey et al., 2012; Tseng et al., 2017).

Silibinin produces epigenetic and EMT regulatory effects that differ according to experimental models. It alters PRC2 and HDAC expression and histone modifications in prostate cancer and NSCLC cells while suppressing ZEB1/SLUG-associated EMT (Wu et al., 2010; Mateen et al., 2012; Anestopoulos et al., 2016). It also enhances gefitinib or erlotinib activity in T790M-positive NSCLC models and suppresses STAT3-driven crizotinib resistance in ALK-rearranged cells (Rho et al., 2010; Cuyàs et al., 2016). In NSCLC cells with ALK rearrangement that are resistant to crizotinib, silibinin inhibits TGF-β/SMAD signaling and restores responsiveness to brigatinib and lorlatinib (Verdura et al., 2022). In MCF-7 breast cancer cells that develop resistance to tamoxifen, EGCG also suppressed Nrf2 signaling and partially restored tamoxifen sensitivity (Esmaeili, 2016).

Non-coding RNAs provide a potential link between flavonoid-induced epigenetic regulation and treatment response. In breast cancer cells, luteolin increased miR-203 expression and suppressed EMT-associated signaling, suggesting that flavonoid-regulated miRNAs may influence phenotypes associated with invasion and therapeutic tolerance (Gao et al., 2019). Genistein upregulated miR-34a and suppressed the oncogenic lncRNA HOTAIR in castration-resistant prostate cancer cells, contributing to reduced proliferation, migration, and invasion; however, the study did not evaluate restoration of endocrine therapy sensitivity (Chiyomaru et al., 2013). A quercetin–hyperoside combination suppressed miR-21 and restored PDCD4 expression in PC3 prostate cancer cells (Yang et al., 2015). In 143B osteosarcoma cells, quercetin enhanced cisplatin sensitivity by upregulating miR-217 and suppressing KRAS, with miR-217 knockdown attenuating this effect (Zhang X. et al., 2015). However, these effects are highly dependent on the compound, tumor model, treatment exposure, and baseline epigenetic state.

More broadly, flavonoids may reverse epigenetic-associated drug resistance by coordinating DNA methylation, histone modification, and non-coding RNA networks. Through these effects, they can reshape drug efflux, EMT, apoptosis, and survival signaling. Epigenetic regulation, however, is highly context-dependent and varies with tumor type, dose, exposure time, and baseline epigenetic state. Methylome profiling, chromatin accessibility analysis, and RNA-network validation are needed to distinguish causal epigenetic events from secondary responses. The principal resistance-reversing mechanisms involving drug efflux, EMT, and epigenetic remodeling are integrated in Figure 4.

**FIGURE 4:**
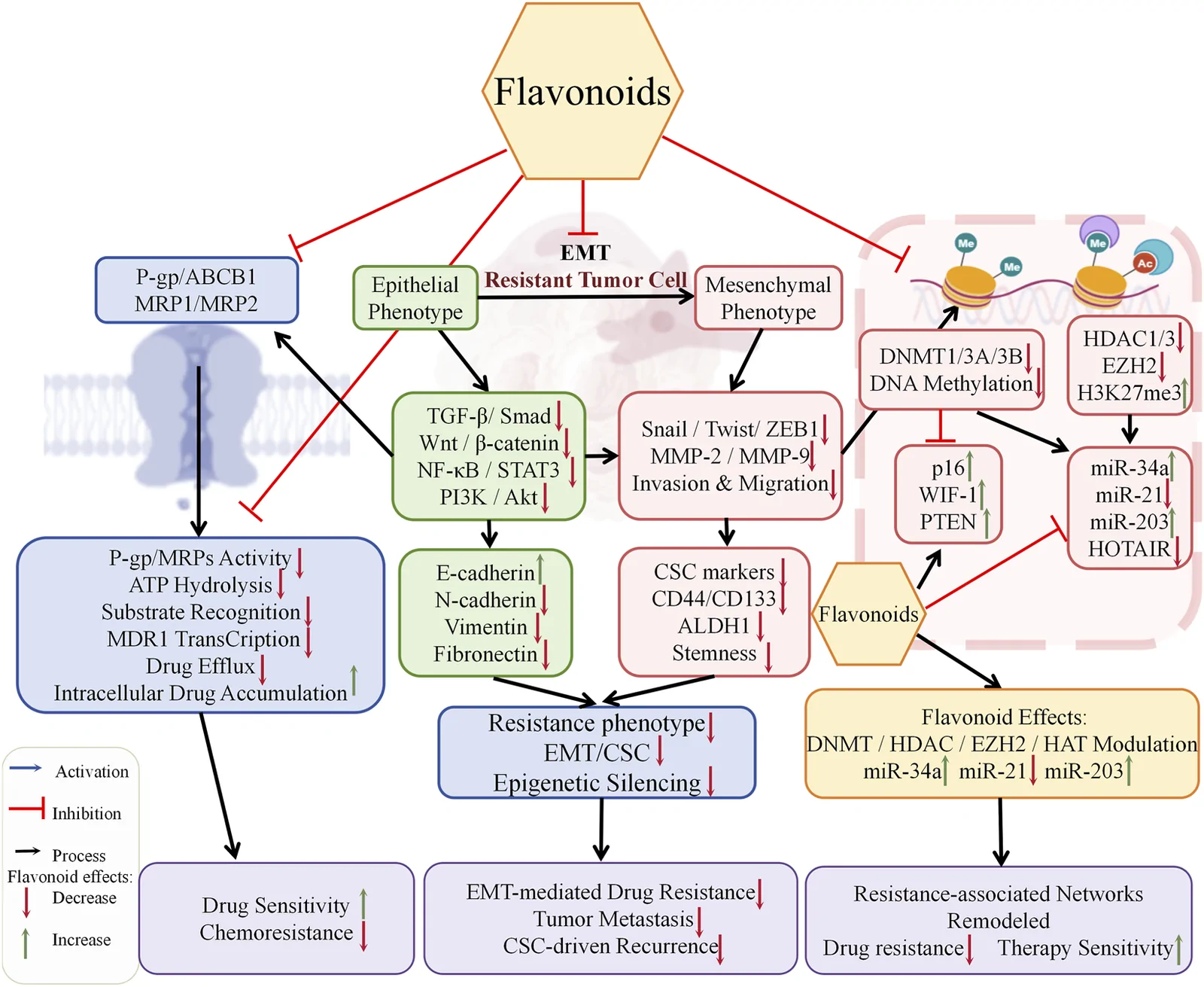
Flavonoid-mediated reversal of cancer therapy resistance through drug efflux inhibition, EMT suppression, and epigenetic remodeling.

## 5 Emerging therapeutic strategies

### 5.1 Combination therapy strategies

Cancer drug resistance is multifactorial and involves drug efflux, DNA repair, apoptosis resistance, EMT, and microenvironmental adaptation (Roszkowska, 2024; Tufail et al., 2024). Adaptation across these mechanisms can limit durable responses to single-agent treatment. Flavonoids have therefore been explored in combination with chemotherapy, targeted therapy, and radiotherapy (Zhai et al., 2021; Renault-Mahieux et al., 2022). These combinations are intended to increase treatment sensitivity, reverse resistant phenotypes, or reduce treatment-related toxicity, but each objective requires separate experimental validation.

Co-delivery systems or combination therapy built around flavonoids may enhance antitumor efficacy by improving drug sensitivity and limiting resistance (Renault-Mahieux et al., 2022; Yadav et al., 2023). Nanocarrier-based co-encapsulation is particularly useful because it can help maintain a predefined drug ratio during delivery (Renault-Mahieux et al., 2022). For example, quercetin and doxorubicin co-encapsulated in biotin-modified PEG–PCL nanoparticles enhanced chemosensitivity in resistant breast cancer cells (Lv et al., 2016). This effect was associated with inhibition of P-gp-mediated drug efflux and suppression of PI3K/Akt signaling (Lv et al., 2016).

Baicalein and paclitaxel co-loaded in nanoemulsions also showed improved activity against paclitaxel-resistant breast cancer (Meng et al., 2016). This combination enhanced antitumor efficacy by increasing oxidative stress, reducing P-gp expression, and producing a higher tumor inhibition rate than either drug alone (Meng et al., 2016). Quercetin enhanced the activity of 5-fluorouracil (5-FU) in colorectal cancer models by suppressing 5-FU-induced thymidylate synthase expression and promoting p53-associated mitochondrial dysfunction (Yang C. et al., 2022). In 5-FU-resistant HCT116 cells, the combination reduced proliferation and increased apoptosis, supporting a preclinical resensitizing effect (Yang C. et al., 2022).

Combining flavonoids with conventional anticancer agents has improved treatment responses in a range of preclinical models. Apigenin enhanced cisplatin-induced DNA damage and apoptosis in several p53-competent cancer cell lines by promoting MAPK-dependent p53 phosphorylation and accumulation (Liu et al., 2017). Separately, apigenin alleviated cisplatin-induced renal oxidative stress, inflammation, and functional injury in non-tumor-bearing mice (Hassan et al., 2017). Phloretin combined with sorafenib showed enhanced antitumor activity in sorafenib-resistant hepatocellular carcinoma models (Saraswati et al., 2019). *In vivo*, this combination increased tumor growth inhibition from 51%–54% to 80%–89%, without obvious systemic toxicity (Saraswati et al., 2019). Myricetin also reversed cisplatin resistance in hepatocellular carcinoma by suppressing YAP signaling (Li M. et al., 2019). Combined treatment with myricetin and cisplatin further inhibited tumor cell proliferation and promoted apoptosis *in vitro* and in xenograft models (Li M. et al., 2019).

Although flavonoid-based combinations may enhance anticancer activity, their safety requires equal attention (Chan et al., 2023). Flavonoids can modulate CYP enzymes, UGTs, P-glycoprotein, BCRP, and other drug transporters. These effects may alter the absorption, distribution, metabolism, and elimination of co-administered anticancer drugs (Li and Paxton, 2013; Miron et al., 2017). Increased intracellular or systemic drug exposure may improve efficacy, but it may also increase normal-tissue exposure and toxicity (Chan et al., 2023). Therefore, an apparent enhancement of antitumor activity may partly reflect altered pharmacokinetics rather than true pharmacodynamic synergy.

Dose dependence represents another source of uncertainty. Flavonoids can show biphasic or hormetic responses and may shift between antioxidant and prooxidant effects as concentration and cellular conditions change (Vazhappilly et al., 2025). Combination treatment may therefore change both the magnitude and direction of their redox effects. This issue may be especially important when the partner drug also induces oxidative stress or DNA damage. Overlapping pharmacological effects could improve tumor killing but may also narrow the therapeutic window (Golonko et al., 2022). Future studies should therefore evaluate efficacy and safety together. Quantitative interaction analyses should be combined with pharmacokinetic and pharmacodynamic measurements (Guan et al., 2025). Dose ratios, administration schedules, plasma and tumor exposure, active metabolites, normal-tissue toxicity, and clinically achievable concentrations should all be considered (Paller et al., 2014; Guan et al., 2025). Such studies are needed to distinguish true pharmacodynamic synergy from exposure-driven enhancement and to define combination regimens with an acceptable therapeutic window.

### 5.2 Nanomedicine and delivery systems

Although many flavonoids show anticancer activity in experimental models, their clinical translation remains limited by poor water solubility, low bioavailability, rapid metabolism, and limited *in vivo* stability (Caro et al., 2022; Ferreira et al., 2022; Farhan et al., 2023; Sysak et al., 2023). These properties reduce systemic exposure and make it difficult to maintain effective concentrations at tumor sites (Caro et al., 2022; Sysak et al., 2023).

Nanodelivery systems may address some of these limitations by improving flavonoid solubility, stability, bioavailability, or tumor delivery (Khan et al., 2021; Ferreira et al., 2022). Depending on their formulation, they can also modify drug release, circulation time, and tumor accumulation (Campani et al., 2022; Caro et al., 2022). These properties support further investigation of nanomedicine as a delivery approach for selected flavonoid-based formulations.

Several delivery systems have been investigated to improve flavonoid bioavailability and stability. Quercetin-loaded poly(lactic-co-glycolic acid) nanoparticles, chitosan–alginate nanocomposites, and liposomes can improve water solubility and intestinal absorption (Caro et al., 2022). They may also prolong circulation and increase passive accumulation in tumor tissue (Caro et al., 2022). Surface-functionalized Fe_3_O_4_ magnetic nanoparticles containing EGCG show improved *in vivo* stability (Sysak et al., 2023). Their pH-responsive release profile allows drug release in the acidic tumor microenvironment, which may reduce exposure in normal tissues (Sysak et al., 2023).

However, nanomedicine is not a complete solution. Particle size, surface charge, drug loading, release kinetics, immune compatibility, and long-term toxicity all affect therapeutic outcomes. The enhanced permeability and retention effect also varies across tumors and patients. Future delivery systems should therefore be evaluated for pharmacokinetics, biodistribution, tumor penetration, and safety.

## 6 Discussion

Flavonoids influence multiple cancer-associated processes, including regulated cell death, oncogenic signaling, metabolic adaptation, tumor microenvironment remodeling, and therapy resistance. This broad activity provides a rationale for their development as adjunctive agents in cancer therapy. However, most current evidence remains preclinical, and translation is limited by pharmacokinetic constraints, tumor heterogeneity, model dependence, and potential safety concerns (Kang et al., 2026; Shoheeduzzaman et al., 2026).

The anticancer effects of flavonoids are better understood as network-level regulation than as isolated single-target actions. Redox and metabolic changes influence cell death competence, while oncogenic signaling shapes stromal and immune responses. These processes also affect drug transport, epithelial–mesenchymal plasticity, and epigenetic adaptation. The pharmacological advantage of flavonoids may therefore arise from coordinated effects across several connected tumor-supporting networks.

Recent reviews have addressed complementary aspects of this field. Dubey et al. emphasized pharmacokinetics, drug-likeness, toxicity, and formulation-based translation, while also summarizing selected anticancer mechanisms (Dubey et al., 2025). Zhu et al. focused on the immunomodulatory actions of natural products in platinum and other forms of treatment resistance (Zhu et al., 2026). The present review instead links regulated cell death, oncogenic and metabolic signaling, the tumor microenvironment, resistance mechanisms, and structure–activity relationships within a flavonoid-centered framework.

A major limitation is the generally poor pharmacokinetic performance of many flavonoids. Compounds such as quercetin and EGCG often exhibit low water solubility, incomplete intestinal absorption, extensive phase II metabolism, and rapid elimination (Manach et al., 2005; Thilakarathna and Rupasinghe, 2013). Consequently, dietary intake or conventional oral administration may not generate tissue exposures comparable to the nominal concentrations used *in vitro*. Pharmacological interpretation should therefore consider not only the parent aglycones but also glucuronidated, sulfated, or methylated conjugates and microbiota-derived metabolites, which may differ in distribution and biological activity (Noh et al., 2016; Cassidy and Minihane, 2017; Zhang et al., 2019). Baicalin, for example, undergoes microbiota-mediated hydrolysis before absorption and subsequent reconjugation, illustrating how intestinal metabolism can alter the active species reaching systemic tissues (Noh et al., 2016).

The gut microbiota is another important factor in the clinical translation of flavonoids. Many flavonoids undergo microbial biotransformation after oral administration, generating metabolites whose bioavailability and biological activity may differ from those of the parent compounds (Al-Ishaq et al., 2021). Microbial metabolism should not, however, be regarded as an obligatory activation step for all flavonoids. Parent compounds or absorbable aglycones can exert direct pharmacological effects in cell-based models. For some flavonoids, microbial transformation can increase the availability of specific chemical forms or generate additional bioactive metabolites (Osborn et al., 2021; Oliveira et al., 2026). Their activities may be weaker, comparable, or stronger than those of the parent compounds, depending on structure and biological context (Wang et al., 2023). Flavonoids can also alter gut microbial communities and secondary microbial metabolites, including short-chain fatty acids and bile acids. These changes may influence host metabolism, immune responses, treatment efficacy, and toxicity (Alexander et al., 2017; Al-Ishaq et al., 2021; Luu et al., 2023). Fecal microbiota transplantation studies suggest that part of a flavonoid-associated phenotype can be transferred through the microbiota, but this does not establish the microbiota as the sole mediator (Zhu et al., 2024). Some changes may weaken after flavonoid withdrawal, indicating dependence on continued exposure (Tzounis et al., 2011). Distinguishing these contributions requires identification of the chemical species reaching relevant tissues, comparison of parent compounds and metabolites at physiologically relevant concentrations, and integration of microbiota depletion with pharmacokinetic profiling and metabolite-replacement experiments (Williamson et al., 2018).

These findings raise an important pharmacological question: whether the *in vivo* effects attributed to flavonoids are mediated mainly by the parent compounds, host-derived conjugates, microbial metabolites of flavonoids, microbiota-derived signaling metabolites, or a combination of these components. Their relative contributions remain poorly defined (Xiong et al., 2023). This uncertainty is further complicated by substantial interindividual variation in gut microbiota composition and metabolic capacity (Xiong et al., 2023). Different individuals may therefore generate distinct metabolite profiles and achieve different systemic exposures after receiving the same flavonoid (Favari et al., 2024). Such variation may contribute to heterogeneous therapeutic responses. Future studies should combine microbiome profiling with metabolomics and pharmacokinetic measurements of parent flavonoids, host conjugates, and major microbial metabolites. Microbiota depletion, fecal microbiota transplantation, and metabolite replacement experiments may further help distinguish direct flavonoid effects from microbiota-mediated effects. Identifying microbiota-defined metabolic phenotypes may ultimately improve the interpretation of treatment variability and support patient stratification.

Chemical structure is a major determinant of this network activity. Hydroxylation, methylation, glycosylation, ring saturation, and other substitutions can alter redox behavior, membrane permeability, metabolism, transporter interactions, and target engagement (Cai et al., 2009). Closely related flavonoids may therefore produce different effects across tumor models. Chemical optimization should improve exposure without disrupting the molecular features required for pharmacological activity (Dong X. et al., 2023; Li et al., 2023).

The antioxidant and anti-inflammatory properties of flavonoids are similarly context dependent. In non-malignant or inflamed tissues, these compounds may reduce oxidative damage and inflammatory signaling. In tumor cells with altered metabolism and redox homeostasis, however, some flavonoids can increase ROS, disrupt glutathione-dependent defenses, alter iron availability, or promote lipid peroxidation. Naringenin, for example, protected lung epithelial cells against ferroptotic injury but enhanced the response of liver cancer cells to ferroptosis inducers in a different experimental setting (Fan et al., 2024; Li et al., 2024). Luteolin-induced ferroptosis has also been supported in renal and prostate cancer models through iron-dependent mechanisms and functional rescue experiments (Han et al., 2022; Fu et al., 2024). Conversely, quercetin protected non-malignant breast epithelial cells during irradiation but did not provide equivalent protection to breast cancer cells (Li et al., 2026). It also reduced doxorubicin-associated oxidative injury in vascular tissue while partially weakening doxorubicin activity in breast cancer cells (Henidi et al., 2020). These findings indicate that antioxidant capacity alone cannot predict the effect of a flavonoid in tumors or during combination therapy.

The gap between experimental models and clinical application remains substantial. Many mechanistic studies use cancer cell lines exposed to concentrations that may not be achievable *in vivo*, while animal studies do not always evaluate tumor exposure, active metabolites, dose schedules, or long-term safety. Drug combinations require particular caution because flavonoids can modulate CYP enzymes, UGTs, P-gp, BCRP, and other transporters, thereby altering the exposure, efficacy, or toxicity of anticancer agents (Kale et al., 2008). Their effects may also differ across tumor types, molecular subtypes, metabolic states, and microenvironmental conditions. Enhanced experimental activity should therefore not be interpreted as clinical benefit without quantitative interaction analysis and adequate pharmacokinetic and safety assessment.

Future studies should integrate clinically relevant exposure levels with pharmacokinetic, metabolomic, microbiome, immune, and tumor molecular analyses. Comparisons between malignant and matched non-malignant models, together with functional measurements of oxidative stress and cell death rescue, would help distinguish tumor-selective activity from nonspecific toxicity or tumor-protective antioxidant effects. Emerging concepts such as mitoxyperiosis and mitoxyperilysis may offer hypotheses linking mitochondrial stress, innate immunity, and lytic cell death, but they remain future research directions rather than established mechanisms of flavonoid action. Overall, better alignment of compound exposure, active molecular species, tumor context, and functional endpoints will be essential for defining which flavonoids, formulations, and therapeutic combinations have realistic translational potential.

## 7 Conclusion

Flavonoids modulate multiple cancer-associated processes, including regulated cell death, oncogenic signaling, metabolic adaptation, tumor microenvironment remodeling, angiogenesis, and therapy resistance. Their pharmacological effects are compound- and context-dependent, and may vary with chemical structure, dose, tumor type, cellular redox state, and treatment schedule. Current evidence therefore supports the continued investigation of structurally defined flavonoids primarily as candidate adjunctive or sensitizing agents rather than established stand-alone anticancer therapies.

A priority for future work is to integrate structure-guided compound selection with evaluation of drug transport, EMT-associated plasticity, and epigenetic adaptation. Nanodelivery may improve exposure, but its benefits require pharmacokinetic, biodistribution, and safety validation. Studies should use clinically relevant concentrations, resistant and matched non-malignant models, active-metabolite profiling, and functional mechanism assays. Mitoxyperiosis represents a testable research direction linking mitochondrial stress with inflammatory cell death, but it has not been established as a flavonoid-regulated mechanism. Defining reproducible relationships among flavonoid structure, tumor exposure, network activity, and treatment response will be essential for evaluating clinical translation.

## Glossary

AKT: Protein kinase B
AP-1: Activator protein 1
ABC: ATP-binding cassette
AMPK: AMP-activated protein kinase
BCRP: breast cancer resistance protein
CAFs: cancer-associated fibroblasts
CSCs: Cancer stem cells
CYP: cytochrome P450
DAMPs: damage-associated molecular patterns
DNMT: DNA methyltransferase
EGCG: epigallocatechin-3-gallate
EMT: epithelial–mesenchymal transition
ERK: Extracellular signal-regulated kinase
FDA: Food and Drug Administration
GPX4: glutathione peroxidase 4
GSH: glutathione
HDAC: histone deacetylase
HIF-1α: hypoxia-inducible factor 1α
MAPK: Mitogen-activated protein kinase
MDR: multidrug resistance
MDSCs: myeloid-derived suppressor cells
MRP1: multidrug resistance-associated protein 1
NF-κB: nuclear factor kappa B
NLRP3: NLR family pyrin domain containing 3
NSCLC: non-small-cell lung cancer
PCD: programmed cell death
PI3K: Phosphatidylinositol 3-kinase
PD-L1: programmed death-ligand 1
P-gp: P-glycoprotein
RCD: regulated cell death
ROS: reactive oxygen species
SAR: structure–activity relationship
TAMs: tumor-associated macrophages
TME: tumor microenvironment
UGTs: UDP-glucuronosyltransferases
VEGF: Vascular endothelial growth factor
